# Metabolic engineering of Clostridium autoethanogenum for selective alcohol production

**DOI:** 10.1016/j.ymben.2017.01.007

**Published:** 2017-03

**Authors:** Fungmin Liew, Anne M. Henstra, Michael Kӧpke, Klaus Winzer, Sean D. Simpson, Nigel P. Minton

**Affiliations:** a BBSRC/EPSRC Synthetic Biology Research Centre (SBRC), School of Life Sciences, University Park, The University of Nottingham, Nottingham NG7 2RD, UK; b LanzaTech Inc., 8045 Lamon Avenue, Suite 400, Skokie, IL, USA

**Keywords:** Gas fermentation, Metabolic engineering, Aldehyde:ferredoxin oxidoreductase (AOR), Bi-functional aldehyde/alcohol dehydrogenase (AdhE), *Clostridium autoethanogenum*

## Abstract

Gas fermentation using acetogenic bacteria such as *Clostridium autoethanogenum* offers an attractive route for production of fuel ethanol from industrial waste gases. Acetate reduction to acetaldehyde and further to ethanol via an aldehyde: ferredoxin oxidoreductase (AOR) and alcohol dehydrogenase has been postulated alongside the classic pathway of ethanol formation via a bi-functional aldehyde/alcohol dehydrogenase (AdhE). Here we demonstrate that AOR is critical to ethanol formation in acetogens and inactivation of AdhE led to consistently enhanced autotrophic ethanol production (up to 180%). Using ClosTron and allelic exchange mutagenesis, which was demonstrated for the first time in an acetogen, we generated single mutants as well as double mutants for both *aor* and *adhE* isoforms to confirm the role of each gene. The *aor1+*2 double knockout strain lost the ability to convert exogenous acetate, propionate and butyrate into the corresponding alcohols, further highlighting the role of these enzymes in catalyzing the thermodynamically unfavourable reduction of carboxylic acids into alcohols.

## Highlights

• 180% improvement in *C. autoethanogenum* ethanol production via metabolic engineering.
• Confirmed role of AOR in autotrophic ethanol production of acetogens.
• Generated both *aor* and *adhE* mutants of *C. autoethanogenum.*.
• Demonstrated allelic exchange mutagenesis for stable deletions in acetogens.
• Inactivation of *adhE* and *aor2*, but not *aor1*, improves autotrophic ethanol production.

## 1 Introduction

The deleterious environmental impact caused by the continuing extraction and exploitation of fossil fuels for energy, coupled with their inherent finite nature, are the principle drivers for the development of sustainable alternatives. One option is to develop a biological route. However, the economic conversion of non-food, cellulosic feedstocks into liquid transportation fuels through biological fermentation is proving challenging. Alternative conversion processes are required. In this regard, gas fermentation has emerged as a promising technology that converts industrial waste gases or syngas containing CO, CO_2_ and H_2_ into fuels without impacting on food production. It is reliant on bacterial process organisms that are able to utilise single carbon gases as a source of carbon typified by a group of strictly anaerobic bacteria known as acetogens. One such acetogen is *Clostridium autoethanogenum* (Abrini et al., 1994). During autotrophic growth, *C. autoethanogenum* employs the Wood-Ljungdahl pathway to fix CO_2_, together with H_2_ as reductant, into predominantly acetic acid and ethanol. It is also able to grow on CO as a sole source of carbon and energy and synthesize ethanol, 2,3-butanediol and lactate (Köpke et al., 2011).

Insight into the metabolic capabilities of *C. autoethanogenum* has been gleaned from the determination of its genome sequence (Brown et al., 2014, Humphreys et al., 2015, Utturkar et al., 2015) and ‘omics’ data (Köpke et al., 2011, Marcellin et al., 2016, Mock et al., 2015) under different growth conditions (fructose, CO and H_2_+CO_2_). Moreover, some understanding of how the acetogen conserves energy while generating reduced products ethanol and 2,3-butanediol autotrophically was derived through the determination of the specific activities and cofactor specificities of all relevant oxidoreductases from cell extracts (Mock et al., 2015, Wang et al., 2013). As yet, however, the biosynthetic capabilities of *C. autoethanogenum*, and in particular ethanol synthesis, have not been thoroughly investigated at the genetic and molecular levels.

**Fig. 1:**
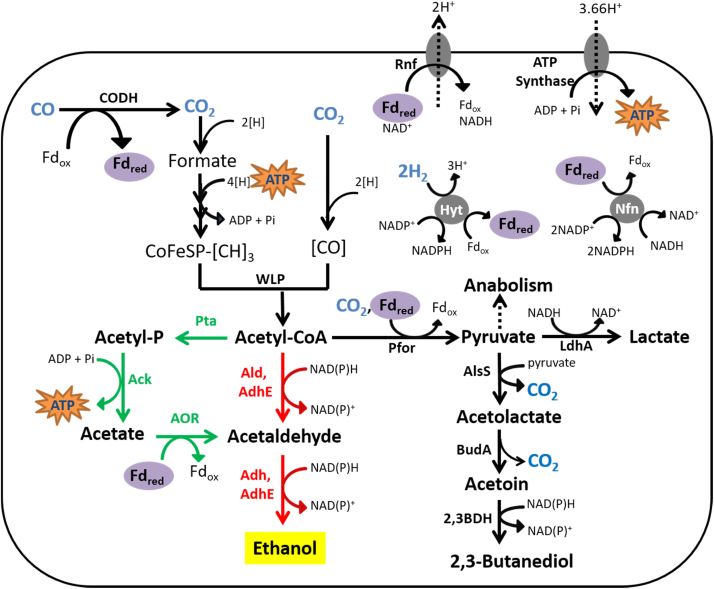
Autotrophic product formation in *C. autoethanogenum*. The ATP-efficient, indirect ethanol route employing phosphotransacetylase (Pta), acetate kinase (Ack) and aldehyde:ferredoxin oxidoreductase (AOR) are depicted in green. The direct ethanol biosynthesis route utilizing bi-functional aldehyde/alcohol dehydrogenase (AdhE), CoA-dependent acetaldehyde dehydrogenase (Ald) and alcohol dehydrogenase (Adh) is shown in red. AlsS = acetolactate synthase; 2,3-BDH =2,3-butanediol dehydrogenase; BudA = acetolactate decarboxylase; CODH = carbon monoxide dehydrogenase; CoFeSP = corrinoid iron sulphur protein; Fd_ox_ = oxidized ferredoxin; Fd_red_ = reduced ferredoxin; Hyt = NADP-dependent electron bifurcating hydrogenase; LdhA = lactate dehydrogenase; Nfn = transhydrogenase; Pfor = pyruvate:ferredoxin oxidoreductase; Rnf = H^+^-translocating ferredoxin: NAD^+^-oxidoreductase; WLP = Wood-Ljungdahl Pathway. (For interpretation of the references to color in this figure legend, the reader is referred to the web version of this article.)

Similar to other prominent autotrophic ethanol producers (e.g., *Clostridium ljungdahlii*, “*Clostridium ragsdalei*”, and *Clostridium carboxidivorans*), the ethanol biosynthesis pathway of *C. autoethanogenum* comprises two main routes (Fig. 1): (i) the direct, two-step sequential reduction of acetyl-CoA into ethanol via acetaldehyde using bi-functional aldehyde/alcohol dehydrogenase (AdhE), CoA-dependent acetaldehyde dehydrogenase (Ald) and alcohol dehydrogenase (Adh) as found in other ethanol producing bacteria including *E. coli* (Membrillo-Hernandez and Lin, 1999) and; (ii) a postulated indirect route that proceeds via acetate and employs aldehyde: ferredoxin oxidoreductase (AOR) to first reduce acetic acid to acetaldehyde before ethanol synthesis via Adh (Köpke et al., 2010, Mock et al., 2015). The genome of *C. autoethanogenum* contains genes encoding two AOR isoforms: *aor1* (CLAU_0081) and *aor2* (CLAU_0099); and two AdhE enzymes: *adhE1* (CLAU_3655) and *adhE2* (CLAU_3656) that appear in tandem and are potentially a result of gene duplication (Humphreys et al., 2015). The same arrangement is also found in *C. ljungdahlii* (Köpke et al., 2010, Leang et al., 2013).

One key distinction between the two ethanol biosynthesis routes is that the indirect route reduces acetate, which is an unwanted by-product as it limits yield and is known to be toxic at elevated concentrations. All naturally isolated acetogens form acetate as it provides an advantage through conservation of one ATP per mole of acetate via substrate level phosphorylation (SLP), which is significant under the ATP-limiting conditions of autotrophic growth. Thermodynamic and stoichiometric analyses estimated that during autotrophic growth of *C. autoethanogenum* on H_2_ + CO_2_, the ATP yield is only 0.5 ATP/mol ethanol via acetyl-CoA reduction to acetaldehyde, in comparison to the 1.2 ATP/mol ethanol via acetic acid reduction to ethanol (Mock et al., 2015). Similarly, during growth on CO, ATP yield would be greater via the AOR route (1.875 ATP/mol ethanol) compared to the AdhE route (1.375 ATP/mol ethanol) when applying the same scheme.

In this study, the *adhE* and *aor* genes of *C. autoethanogenum* were inactivated to determine their roles in autotrophic ethanol production. In addition to the intron-based gene inactivation demonstrated previously (Marcellin et al., 2016, Mock et al., 2015), here we expanded the genetic tools for this acetogen to include allelic exchange. Characterization of these strains revealed the roles of AOR in supporting alcohol production, and demonstrated that strains producing greater amounts of ethanol (up to 180% improvement) could be obtained by genetically inactivating *adhE* or *aor2*.

## 2 Materials and methods

### 2.1 Bacterial strains and growth conditions

The bacterial strains used are listed in Table S1. *Escherichia coli* strains employed for general plasmid propagation, cloning and conjugation were cultivated at 37 °C in LB medium in the presence of antibiotic (25 μg/mL chloramphenicol, 100 μg/mL spectinomycin). *C. autoethanogenum* DSM 10061 was purchased from Deutsche Sammlung von Mikroorganismen und Zellkulturen (DSMZ) GmbH, Braunschweig, Germany and routinely cultivated under strict anaerobic conditions in CaGM medium (Liew et al., 2016a).

Cell growth on liquid medium was monitored spectrophotometrically at 600 nm (OD_600_). Changes in headspace pressure were measured using Rugged Digital Pressure Gauge DPG120 (Omega Engineering). For growth of *C. autoethanogenum* on agar plates, YTF solid medium (10*g*/L fructose, 10*g*/L yeast extract, 16*g*/L tryptone, 0.2*g*/L sodium chloride, 15*g*/L bacteriological agar (oxoid), pH 5.8), with antibiotics (7.5 μg/mL thiamphenicol, 6 μg/mL clarithromycin) where appropriate, was used. All mutagenesis work was performed inside an anaerobic workstation at 37 °C (Don Whitley Scientific Ltd). For strain comparisons, 3–4 biological replicates containing *C. autoethanogenum* wild-type (WT) or recombinant strains were grown in 250 mL serum bottles containing 50 mL CaGM medium with either 10*g*/L fructose, 200 kPa CO, or 150 kPa H_2_ +50 kPa CO_2_ as growth substrate. Incubation at 37 °C was undertaken with agitation (225 rpm) inside New Brunswick Innova shakers (Eppendorf). A standardized 0.5 OD_600_ equivalent of exponentially growing cultures were used as inoculum. For instance, 0.25 mL of a pre-culture with OD_600_ of 2 would be used as inoculum.

### 2.2 DNA manipulations

Genomic DNA from *C. autoethanogenum* was isolated using DNeasy Blood and Tissue kit (Qiagen) for PCR diagnostics. For Southern Blot analysis, genomic DNA of *C. autoethanogenum* was extracted according to Bertram and Dürre (1989). Plasmid DNA from *C. autoethanogenum* was isolated using QIAprep Spin Miniprep kit (Qiagen) with the supplementation of 20 mg/mL chicken lysozyme into lysis buffer and incubation at 37 °C for 30 min before proceeding to downstream procedures. Polymerase Chain Reaction (PCR) was carried out using Phusion DNA polymerase (NEB) or Q5 DNA polymerase (NEB). All primers used in this study are listed in Table S2. Primers were designed using Geneious 6.1.7 (Biomatters) and synthesized by Sigma-Aldrich or Eurofins. Sanger sequencing of plasmids and amplicons was carried out by Source Bioscience Plc.

### 2.3 Plasmid vectors and allelic-exchange cassettes

All plasmids used in this study are derived from the pMTL80000 series of modular, *E. coli-Clostridium* shuttle vectors (Heap et al., 2009) and are listed in Table S3. For the construction of plasmid ‘pMTL83151-P_acsA_’, the promoter region of *acsA* (CLAU_1579) of *C. autoethanogenum* was amplified using oligonucleotides ‘P_acsA_-*Not*I-F’ and ‘P_acsA-_*Nde*I-R’ followed by cloning into plasmid pMTL83151 (Heap et al., 2009) using restriction sites *Not*I and *Nde*I. To construct the *aor1* expression plasmid, ‘pMTL83151-P_acsA_-aor1’, *aor1* was subjected to two rounds of splice-overlapping extension (SOE-PCR) (Warrens et al., 1997) using primers listed in Table S2 to remove two interfering *Nde*I sites before cloning using restriction sites *Nde*I and *Kpn*I. At both interfering sites (nucleotide 975 and 1284), nucleotides ‘CATATG’ were mutated to ‘CTTATG’ coding for the same amino acids. For the construction of ClosTron retargeting plasmids, the appropriate intron targeting regions within *adhE1*, *adhE2*, *aor1* and *aor2* were generated *in silico* from www.ClosTron.com using the Perutka algorithm (Perutka et al., 2004). DNA 2.0 Inc. then synthesized the 344 bp intron targeting region and cloned it into ClosTron vector pMTL007C-E2 (Heap et al., 2010) using restriction sites *Hin*dIII and *Bsr*GI, resulting in plasmids ‘pMTL007C-E2::adhE1a_115 s’ (targeting upstream Ald domain of *adhE1*), ‘pMTL007C-E2::adhE1b_541 s’ (targeting downstream Adh domain of *adhE1*), ‘pMTL007C-E2::adhE2_662 s’, ‘pMTL007C-E2::aor1_361 s’ and ‘pMTL007C-E2::aor2_370 s’ (Table S3).

An allelic exchange plasmid, ‘pMTL-AMH101’ (Supplementary nucleotide sequence 1), was used for deletion of 227 bp of the C-terminus of *C. autoethanogenum pyrE* (CLAU_1436). It contains a heterologous *pyrE* (cac_0027) from *C. acetobutylicum* ATCC 824 (to be employed as a counter selectable marker) and comprises a 303 bp short left homology arm (LHA) and a larger 1219 bp right homology arm (RHA), with *lacZα* in between, as the allelic-exchange cassette. The in-frame deletion (IFD) allelic-exchange cassettes of *C. autoethanogenum adhE1*, *adhE1+2*, and *aor2* consists of two homology arms of similar lengths (518 – 580 bp), and were assembled using SOE-PCR and oligonucleotides listed in Table S2. All the IFD cassettes retained only the start and stop codons of the target loci without affecting the 5`-untranslated region (UTR) and 3`-UTR. Following SOE-PCR, the IFD cassettes were digested with *Sac*II and *Asc*I and cloned into plasmid pMTL-AMH101 to generate plasmids ‘pMTL84151-∆*adhE1*’, ‘pMTL84151-∆*adhE1+2*’, and ‘pMTL84151-∆*aor2*’. For the restoration of *pyrE*, a plasmid called ‘pMTL-AMH102’ (Supplementary nucleotide sequence 2), which consists of a *pyrE* repair allelic exchange cassette with a 526 bp LHA and 1213 bp RHA, was employed.

### 2.4 Plasmid transfer into *C. autoethanogenum*

Plasmids were transformed into *E. coli* donor strain CA434 (HB101 containing the conjugative plasmid R702) and then transferred into *C. autoethanogenum* via conjugation using previously established methods (Mock et al., 2015, Purdy et al., 2002, Williams et al., 1990). Thiamphenicol (7.5 µg/mL) was used to select for *catP*-based plasmids. Trimethoprim (10 µg/mL) was used to counter select against *E. coli* CA434 after conjugation. For the validation of plasmid complementation strains, plasmids were isolated from *C. autoethanogenum* transconjugants and subsequently transformed into *E. coli* cells, before restriction digest analysis was carried out on the ‘rescued’ plasmids. The 16 s rRNA gene was also amplified from the genomic DNA of transconjugants using oligonucleotides ‘univ-0027-F’ and ‘univ-1492-R’, followed by Sanger sequenced for verification purposes.

### 2.5 Construction of *C. autoethanogenum* ClosTron strains

Following conjugation of ClosTron retargeting plasmids into *C. autoethanogenum*, thiamphenicol and trimethoprim resistant colonies were transferred onto solid YTF medium supplemented with 6 µg/mL clarithromycin to select for Intron insertion in target loci. These were then repeatedly streaked onto the same selective medium until plasmid loss was evident - loss of ability to grow on medium supplemented with thiamphenicol. Genomic DNA was extracted from the clarithromycin resistant colonies and subjected to PCR screen using locus-specific flanking primers (Table S2) to identify clones (Fig. S1A & S2A) that produced an amplicon that is 1.8 kb larger than WT control (indicative of ClosTron insertion at specified DNA locus). Sanger sequencing of the ClosTron amplicons was performed to validate the location of ClosTron insertion. As final verification, Southern Blot analysis was performed using a digoxigenin (DIG) High-Prime DNA labelling and detection kit (Roche) as instructed by the manufacturer to ensure that only one ClosTron insertion had occurred in each mutant (Fig. S1B & S2B).

### 2.6 Allelic-exchange procedure

#### 2.6.1 Creation of ∆*pyrE* strain

The procedure adopted was as previously described (Heap et al., 2012). For the construction of ∆*pyrE* strain, which serves as a host for further IFD of *adhE1*, *adhE1+2*, and *aor2* using *pyrE* as a positive and negative selection marker, the plasmid pMTL-AMH101 was transferred into *C. autoethanogenum* via conjugation. The transconjugants were restreaked on YTF solid medium supplemented with thiamphenicol and trimethoprim to enrich and identify fast-growing single-crossover integrant clones. Genomic DNA was isolated and subjected to PCR analysis using two different primers (ACE-plasmid-F and ACE-plasmid-R) that anneal to plasmid specific sequences together with the appropriate locus-specific flanking primers (Table S2). The presence of a DNA fragment indicated that the clones were indeed single-crossover integrants, while the size was indicative of at which homology arm the recombination event had occurred. PCR verified single-crossover integrants were inoculated into CaGM liquid medium supplemented with 10 g/L fructose and thiamphenicol and allowed to grow for 2 days inside anaerobic workstation, before they were serially diluted and plated. To facilitate the screening of rare second recombination events, the CaGM solid medium had 1 g/L yeast extract replaced with 1 g/L casein acid hydrolysate, and supplemented with 1.5 mg/mL fluoroorotic acid (FOA+) and 5 μg/mL uracil. The efficacy of FOA is reduced by the presence of uracil, and yeast extract contains substantial amounts of uracil so a less enriched supplement such as casein acid hydrolysate was used instead. Incubation at 37 °C was carried out inside anaerobic workstation and FOA-resistant colonies that emerged within 2–3 days were restreaked onto the same selective medium before PCR screen using locus-specific flanking primers was performed to distinguish double-crossover recombinant clones from wild-type revertant clones. Sanger sequencing was employed to confirm the expected genotypes.

#### 2.6.2 Creation of ∆*adhE1*, ∆*adhE1*^mut^, and ∆*adhE1+2* strains

After the loss of plasmid was demonstrated by the loss of thiamphenicol resistance, the ∆*pyrE* strain could serve as a host for pMTL84151-∆adhE1, and pMTL84151-∆adhE1+2 via conjugation for the construction of ∆*adhE1* and ∆*adhE1+2* strains, respectively. Single-crossover integrants and double-crossover FOA-resistant, uracil auxotrophic clones were obtained for both targets (same method as the ∆*pyrE* strain above). In the first attempt, Sanger sequencing revealed that in addition to the IFD of *adhE1*, an unintended 84 bp deletion had occurred in the promoter region of *adhE2*. Termed ‘∆*adhE1*^*mut*^’, this strain also shown to have lost the excised plasmid, as demonstrated by loss of thiamphenicol resistance. A second attempt at generating a ‘clean’ ‘∆*adhE1*’ strain without the unintended 84 bp deletion was successful but repeated attempts to isolate a clone in which the excised plasmid had been lost (shown by persistent thiamphenicol resistance) were unsuccessful. For the ∆*adhE1+2* strain, Sanger sequencing revealed successful deletion of *adhE1* and *adhE2* without complications in the 5`-UTR of *adhE1* and 3`-UTR of *adhE2*. However, repeated restreaking was unable to isolate thiamphenicol sensitive colonies for this strain.

#### 2.6.3 Creation of *aor1+2* double KO strain

For the construction of the *aor1+2* double knock-out strain (herein termed ‘*aor1+2* KO’), the *aor1* locus was first inactivated using ClosTron plasmid pMTL007C-E2::aor1_361 s in a ∆*pyrE* strain. Following the loss of plasmid, the IFD plasmid pMTL84151-∆*aor2* was transformed and the isolation of single-crossover integrant and double-crossover recombinant clones were carried out as described above. These *aor1* and *aor2* double KO but uracil auxotrophic clones were transformed with plasmid pMTL-AMH102 to restore uracil prototrophy. Fast-growing thiamphenicol-resistant colonies were plated onto CaGM solid medium supplemented with 10 g/L fructose but had 1 g/L yeast extract replaced with 1 g/L casein acid hydrolysate without uracil supplementation. As final validation, PCR screening followed by Sanger sequencing was carried out using flanking primers to verify ClosTron insertion event in *aor1*, IFD of *aor2* and restoration of *pyrE*. Plasmid loss was confirmed by demonstration of thiamphenicol sensitivity.

### 2.7 Harvest of cells for gene expression analysis

*C. autoethanogenum* recombinant strains were cultivated in triplicates of 500 mL pressure plus laboratory bottles (Duran), each containing 200 mL CaGM supplemented with 10*g*/L fructose. For strains ∆*pyrE* and ∆*adhE1*^*mut*^*,* 10 µg/mL uracil was supplemented. In order to maintain plasmids in *C. autoethanogenum* harbouring plasmid pMTL83151-P_acsA_ and pMTL83151-P_acsA_-aor1, 7.5 µg/mL of thiamphenicol was supplemented. Approximately 12 OD_600_ equivalent of cells were harvested at various growth phases by centrifugation at 4 °C at 3220×*g* for 10 min. Supernatant was removed and the cell pellet was resuspended in 1 mL RNAlater Stabilization Solution (Ambion) by pipetting. After overnight incubation at 4 °C, the cell suspension was centrifuged at 3220×*g* at 4 °C for 10 min and supernatant discarded before storage at −80 °C until RNA extraction.

### 2.8 Total RNA extraction and cDNA synthesis

Following the addition of 1.5 mL cold TRIzol (Ambion), the thawed cell pellet was transferred into pre-chilled 2 mL microfuge tubes containing 1*g* of dnature 0.1 mm diameter Zirconia/Silica beads (dnature Ltd). Cell disruption was performed in 3 cycles of 1 min bead beating using Mini Beadbeater-16 (dnature Ltd), with 1 min chilling on ice in between the cycles. Following 1 min of 4 °C centrifugation at 20,238×*g*, the supernatant was harvested and 100 μL of chloroform was added, vortexed for 20 s and then incubated at room temperature for 15 min with occasional mixing. After the centrifugation at 20,238×*g* (4 °C) for 15 min, the aqueous phase was collected and 0.7 vol of isopropanol was added. The samples were incubated at room temperature for 10 min before centrifugation at 20,238×*g* (4 °C) for 10 min. Supernatant was removed and the DNA pellet was washed with 700 μL of ice-cold 70% (v/v) ethanol before another round of centrifugation 20,238×*g* (4 °C) for 10 min. Following the removal of supernatant, the RNA pellet was air-dried for 15 min before resuspension in 100 μL of RNase-free water and 1 μL of RNaseOUT (Invitrogen).

Genomic DNA was removed by the addition of TURBO DNase enzyme (Ambion) and 37 °C incubation for 30 min. The DNase-treated RNA was purified using RNA Clean and Concentrator Kit (Zymo Research) as per manufacturer's instructions and stored at −80 °C. The concentration and purity of isolated RNA was analyzed spectrophotometrically using Nanodrop (Thermo Scientific). To ensure the absence of residual genomic DNA in the isolated RNA, 1 μL of each RNA samples was subjected to PCR analysis using primer pairs “adhE2–662s-F” and “adhE2–662s-R”. The quality of RNA was examined using 2100 Bioanalyzer (Agilent Technologies) and RNA samples with RNA integrity number (RIN) greater than 7 were used for cDNA synthesis. Two μg of total RNA was used per 20 μL SuperScript III Reverse Transcriptase reactions (Invitrogen) and diluted 10-fold with RNase-free water prior to qPCR analysis.

### 2.9 Quantitative reverse transcriptase polymerase chain reaction (qRT-PCR)

Primers and probe sets for target gene (*adhE2*) and housekeeping genes (*gyrA* and *rho*) (Table S4) were designed using the Custom TaqMan Assay Design Tool and purchased as Single-Tube Custom TaqMan Gene Expression Assays from Applied Biosystems. *gyrA* (CLAU_2078; encodes DNA gyrase subunit A) and *rho* (CLAU_2269; encodes transcriptional termination factor) were chosen as housekeeping genes because they exhibited the most stable gene expression levels for different carbon sources and stresses in closely related acetogen *C. ljungdahlii* DSM 13528 (Liu et al., 2013). The amplification efficiencies of the TaqMan probes and primers were empirically determined to be between 94.2% and 99.7% (R^2^≥0.998) by constructing a standard curve using serially diluted cDNA as template (data not shown).

All qRT-PCR reactions were set up in 96-well Microseal PCR plates (Bio-Rad Laboratories) and performed in triplicates of 20 μL volume containing 1 μL diluted cDNA, 1 μL of 20x Custom TaqMan Gene Expression Assay, 10 μL of 2x TaqMan Gene Expression Master Mix (Applied Biosystems) and 8 μL nuclease-free water. Non-template controls (NTC) were included for each TaqMan probe and primer qRT-PCR master mixes. Each qRT-PCR runs comprised an initial denaturation and polymerase activation at 95 °C for 12 min, followed by 40 cycles of denaturation at 95 °C for 15 s and combined annealing and extension at 60 °C for 60 s. The CFX connect Real-Time PCR Detection System (Bio-Rad Laboratories) was employed to record the accumulation of signals in each well within the PCR plate, and the accompanying CFX Manager Software was used to perform normalized gene expression analysis.

### 2.10 Analytical chemistry

Analysis of metabolites were performed using Varian ProStar HPLC system equipped with a RID (Refractive Index Detector) operated at 30 °C and an Aminex HPX-87H column (1300×7.8 mm, particle size 9 µm) (Bio-Rad Laboratories) kept at 30 °C. Slightly acidified water was used (0.005 M H_2_SO_4_) as mobile phase with a flow rate of 0.5 mL/min. To remove proteins and other cell residues, samples were centrifuged at 20,238×*g* for 5 min and the supernatant was filtered with Spartan 13/0.2 RC filters. 10 μL of the supernatant was then injected into the HPLC for analyses.

### 2.11 Data analysis and presentations

Statistical analysis and graphically presented results were obtained using GraphPad Prism. Two-tailed, unpaired, parametric student's *t*-tests were employed for comparison of means.

## 3 Results and discussions

### 3.1 Exemplification of allelic-exchange mutagenesis in *C. autoethanogenum*

Whilst single intron insertion mutants of *adhE1*, *adhE2*, *aor1* and *aor2* were readily generated (Fig. S1 & S2), re-use of the ClosTron to generate double mutants requires marker re-cycling (Heap et al., 2010). This proved not to be possible in *C. autoethanogenum* (data not shown). In this study, therefore, we developed an allelic exchange method for *C. autoethanogenum* based on the use of a pseudo-suicide vector reliant on the pCD6 replicon (Heap et al., 2009) and a plasmid-encoded counter selection marker composed of an orotate phosphoribosyltransferase (*pyrE*) gene of *C. acetobutylicum.* The approach taken, and the principles involved, have recently been reviewed (Minton et al., 2016).

**Fig. 2:**
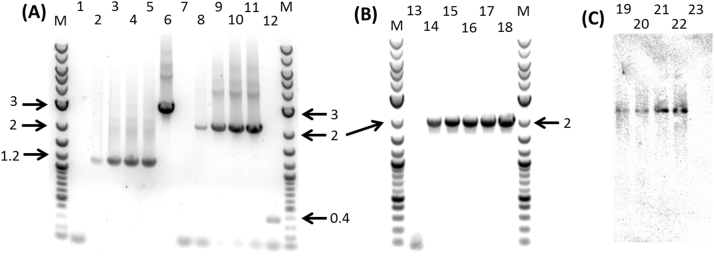
Screening and validation of the *aor* double KO strain with restored *pyrE*. (A) PCR screening of ∆*aor2 and aor1* KO strain; (B) PCR screening of uracil autotrophic *aor* double KO strain for restored *pyrE* allele; (C) Southern Blot analysis of *aor1* KO strain. M = NEB 2-log DNA ladder; 1 – 6= aor2-seq-F and aor2-seq-R primer pair; 7 – 12= aor1–559 s-F and aor1–559 s-R primer pair; 13 – 18= ACE-pyrE-F and ACE-pyrE-R primer pair; 1, 7 and 13= Non-template controls; 6, 12, 18 and 23= *C. autoethanogenum* WT genomic DNA control; 2 – 5, 8 – 11, 14 – 17= clones of *aor* double KO strain with restored *pyrE*; 19 – 22= *Hin*dIII digested genomic DNA of *aor1* KO strain. Arrows and the accompanying numbers denote the fragment sizes of DNA ladder in kilobases.

In order for *pyrE* to be used as a counter selection marker, a *C. autoethanogenum* mutant lacking the 3′-end (227 bp) of the native *pyrE* gene (CLAU_1436) was made using an ACE (Allele-Coupled Exchange) vector equivalent to pMTL-YN18 (Ng et al., 2013), as described in Section 2.3. To create a double mutant, the *aor1* gene was first inactivated using ClosTron mutagenesis in the ∆*pyrE* strain, before IFD of *aor2* was undertaken by allelic exchange using the *pyrE-*based KO vector (pMTL84151-∆aor2) and counter selection using FOA. Following creation of an *aor1+2* KO strain, the mutant *pyrE* allele was restored to WT (uracil prototrophy) using a specially constructed ACE correction vector, analogous to pMTL-YN1 of *C. difficile* (Ng et al., 2013). PCR verification, Sanger sequencing and Southern Blot analysis was performed to confirm the genotype of this strain (Fig. 2).

To explore the consequences of the deletion of both domains of *adhE1*, as well as *adhE1* + *adhE2*, appropriate IFD mutants of *C. autoethanogenum* were sought using *pyrE-*directed allelic exchange. In a first attempt, the strain ∆*adhE1*^mut^ was obtained in which the *adhE1* was deleted but in which 84 bp encompassing the *adhE2* promoter region were also missing (Fig. S3A & D). The deleted *adhE2* 84 bp promoter region was found to be flanked by two 9 bp repeat regions (Fig. S3D). Its deletion most likely placed the *adhE2* gene under the transcriptional control of the stronger *adhE1* promoter (Marcellin et al., 2016, Mock et al., 2015), a hypothesis corroborated by experimental comparison of *adhE2* mRNA levels present in the ∆*adhE1*^mut^ strain and the wildtype (Fig. S10). A second attempt at generating a ‘clean’ IFD strain of *adhE1* without the unintended 84 bp deletion was successful (Fig. S3B). However, the KO plasmid used to make the deletion could not be cured from the resultant ∆*adhE1* strain. Similarly, the KO plasmids used to generate a strain (∆*adhE1+2*) in which both *adhE1* and *adhE2* were successfully deleted (Fig. S3C) could also not be cured. Neither of these strains (∆*adhE1* and ∆*adhE1+2*) were therefore further characterized.

### 3.2 Metabolic engineering of the indirect, AOR ethanol pathway

As the genome of *C. autoethanogenum* encodes two AOR isoforms: *aor1* (CLAU_0081) and *aor2* (CLAU_0099), both single and a double mutant were generated and characterized during autotrophic and heterotrophic growth.

#### 3.2.1 Characterization of single *aor* mutants

**Fig. 3:**
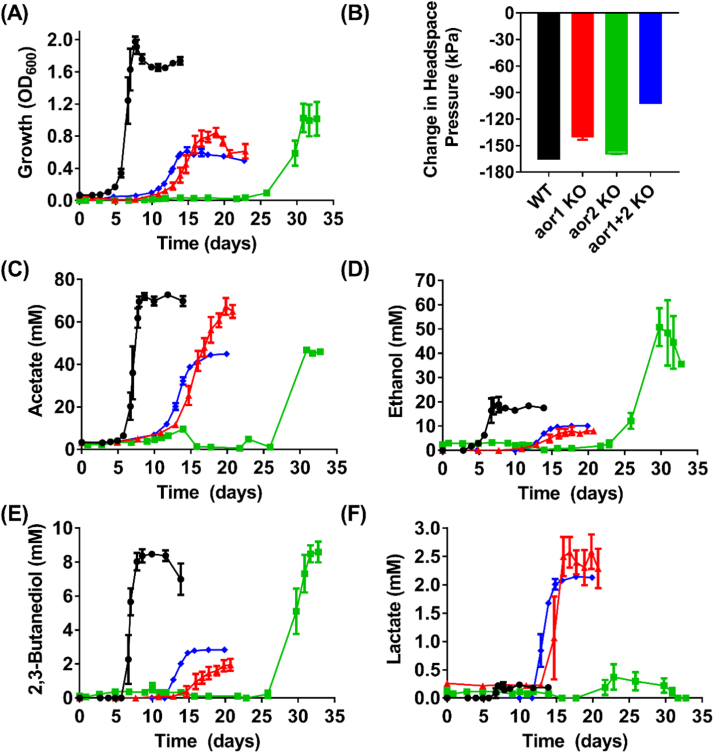
Growth, headspace pressure change and metabolite profiles of *C. autoethanogenum* WT (black circles), *aor1* KO (red triangles), *aor2* KO (green squares), and *aor1+2* KO strains (blue diamonds) on 200 kPa CO. (A) Growth profile; (B) Change in headspace pressure from start to end of cultivation; (C) Acetate profile; (D) Ethanol profile; (E) 2,3-Butanediol profile; and (F) Lactate profile; For each strain n =4, except for *aor2* KO n =3; Error bars = s.e.m. (For interpretation of the references to color in this figure legend, the reader is referred to the web version of this article.)

Whilst the genes encoding the two AOR isozymes are of the same length and share 78% identity, transcriptome data has shown that *aor1* is expressed at 5-10x higher levels than *aor2* during growth on CO (Marcellin et al., 2016, Mock et al., 2015). Both *aor* genes are expressed at higher levels during autotrophic growth as compared to heterotrophic growth (Marcellin et al., 2016). Consistent with the expression data, we found that inactivation of the more highly expressed *aor1* gene restricted the eventual cell density of the mutant culture to half that of the WT (Fig. 3A) when grown on CO and reduced the levels of ethanol and 2,3-butanediol formed to 43% (p-value =0.019) and 23% (*p*-value <0.0001) of the amounts produced by the WT, respectively (Fig. 3D & E). The levels of ethanol produced by the mutant could be restored to those of the WT through the introduction of a complementation plasmid, pMTL83151-P_acsA_-aor1 (Fig. S5). Growth of the *aor1* KO strain was on the other hand not significantly affected on fructose as the carbon source, but ethanol production was reduced 33% (p-value =0.014) compared to the WT (Fig. S6B-D). In contrast, compared to the WT, the inactivation of *aor2* consistently increased ethanol production during growth on CO (Fig. 3) or fructose (Fig. S6), by 170% (*p*-value=0.009) and 47% (*p-*value=0.003), respectively. The ratios of peak ethanol/peak acetate for *aor1* KO strain and *aor2* KO strains are 0.12 and 1.09, respectively, relative to 0.26 in the WT (Table 1). These results collectively suggest a contrasting role between *aor1* and *aor2* in ethanol production in *C. autoethanogenum*.

Both the *aor1* KO and the *aor2* KO strains exhibited (Fig. 3A) a prolonged growth lag phase (10 and 25 days, respectively) and reduced eventual cell density while growing on CO, indicating a deficiency in recycling the reduced ferredoxins generated from CO oxidation. An alternative avenue for the offload of reduced ferredoxin is the reaction involving pyruvate: ferredoxin oxidoreductase (PFOR) that converts acetyl-CoA and CO_2_ to pyruvate, which may subsequently alter the production of pyruvate-derived products such as 2,3-butanediol and lactate (Fig. 1). The 11-fold higher levels of lactate production (Fig. 3F) by the *aor1* KO strain (but not by the *aor2* KO strain) relative to WT indicated that the production of lactate, rather than 2,3-butanediol, is the predominant route for achieving redox balance in the event of *aor1* inactivation. From pyruvate, the generation of lactate involves only one enzyme (lactate dehydrogenase) whereas the biosynthesis of 2,3-butanediol involves three enzymes (acetolactate synthase, acetolactate decarboxylase and 2,3-butanediol dehydrogenase) (Köpke et al., 2014, Köpke et al., 2011) (Fig. 1).

#### 3.2.2 Characterization of double *aor* mutants

During growth on pure CO, the *aor1+2* double KO strain exhibited a prolonged lag phase, eventually achieving a cell density that was 69% lower than the WT (p-value <0.0001) and was only able to reduce the headspace pressure by 101 kPa over the course of the experiment, relative to a decrease of 163 kPa in WT control (Fig. 3A & B). This retarded growth and poor gas consumption highlight the important role of AOR in supporting growth and utilization of CO.

In terms of metabolite production from CO, relative to the WT, the double KO strain produced 46% less ethanol (*p*-value =0.034), 38% less acetate (*p*-value<0.0001), 66% less 2,3-butanediol (*p*-value<0.0001) but 9-fold higher level of lactate (*p*-value<0.0001) (Fig. 3C-F). These results indicate that ethanol could still be synthesized from CO via the direct reduction of acetyl-CoA in the *aor1+2* KO strain.

**Fig. 4:**
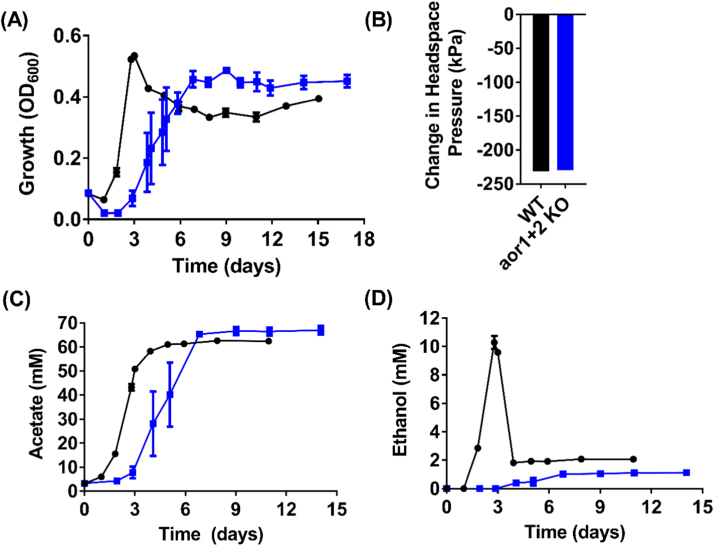
Growth, headspace pressure and metabolite profiles of *C. autoethanogenum* WT, and *aor1+2* KO strain on H_2_+CO_2_. (A) Growth profile; (B) Change in headspace pressure; (C) Acetate profile; (D) Ethanol profile; Black circles = WT (n =4); Blue squares = *aor1+2* KO strain (n =4); Error bars = s.e.m. (For interpretation of the references to color in this figure legend, the reader is referred to the web version of this article.)

On H_2_+CO_2_, the growth lag phase of the *aor1+2* double KO strain slightly increased but was able to grow to similar cell density to the WT and reduced the same amount of headspace pressure as the WT control (Fig. 4A & B). On a molar basis, only half the amount of reduced ferredoxin is generated from H_2_ than CO (Fig. 1), which may lead to less redox imbalance and explain why the KO strain was able to grow largely unaffected on H_2_+CO_2_. Acetate production was not affected but the KO strain produced 9.2-fold less ethanol than WT (p-value <0.0001) (Fig. 4C & D). This is also reflected in the lower peak ethanol/peak acetate ratio of 0.02 in the *aor1+2* KO strain relative to 0.16 in the WT (Table 1). No lactate or 2,3-butanediol was produced by either strain (data not shown).

The finding that a very high specific AOR activity was detected in the cell extract of H_2_+CO_2_–grown *C. autoethanogenum*, which was also 4-fold higher than CO-grown cell extract and 5.3-fold higher than fructose-cultivated cells (Mock et al., 2015), highlighted the significance of AOR in ethanol biosynthesis during H_2_+CO_2_ conditions. Our results confirmed the prediction of Fast and Papoutsakis (2012), Bertsch and Muller (2015), and Mock et al. (2015) that very little ethanol can be generated under the ATP-limiting H_2_+CO_2_ conditions without the action of AOR. Coincidentally, prominent autotrophic ethanol producers such as *C. ljungdahlii* (Köpke et al., 2010) and *C. carboxidivorans* (Bruant et al., 2010) possess AOR whereas non-ethanol producing acetogens such as *Acetobacterium woodii* (Poehlein et al., 2012) lack a functional AOR (Bertsch and Muller, 2015, Mock et al., 2015).

Under ATP-sufficient heterotrophic growth on fructose, the growth, ethanol and 2,3-butanediol production of the *aor1+2* double KO strain was not significantly affected (Fig. S6). In *Pyrococcus furiosus*, the deletion of its only AOR resulted in minimal ethanol production while growing on maltose (Basen et al., 2014).

#### 3.2.3 Carboxylic acid reduction in *aor* double mutant

AOR-harbouring acetogens such as *C. ljungdahlii* and “*C. ragsdalei*” have been shown to catalytically reduce a range of carboxylic acids, such as propionic acid, butyric acid, valeric and caproic acid into the corresponding primary alcohols using CO as electron donor (Isom et al., 2015, Perez et al., 2013). To investigate whether the AOR in *C. autoethanogenum* is capable of catalyzing such reactions, the WT and *aor1+2* double KO strain were subjected to CO growth in the presence of supplemented 60 mM acetate, 40 mM propionate and 40 mM butyrate.

The supplementation of 60 mM acetate (a physiological metabolite) had a stimulatory effect on the CO growth of *C. autoethanogenum* WT as the lag phase was reduced from 5 days to 1 day (Fig. 3A and Fig. S7A), but not the KO strain. Up to 31.3 mM acetate was consumed by the WT during early exponential phase but a net production of 79.1 mM acetate was recorded at stationary phase (Fig. S7C). In the stationary phase, up to 70.8 mM ethanol was generated by the WT (Fig. S7D). In contrast, the *aor*-deficient strain was not able to consume acetate during any of the growth stages and produced only 7.2 mM ethanol (Fig. S7C & D). The reduction of acetic acid to acetaldehyde with reduced ferredoxin is thermodynamically unfavourable under standard conditions (ΔG°′=35 kJ/mol) (Thauer et al., 1977) because of the extremely low potential reaction (E°′=−580 mV) (Loach, 1976). However, at physiological conditions with intracellular pH of 6.0 and 1000-fold higher intracellular acetate than acetaldehyde concentrations, the reaction is exergonic (Mock et al., 2015). The consumption of acetate with concomitant production of ethanol during exponential growth of *C. autoethanogenum* indicates that the acetogen readily catalyzes the reduction of acetic acid using CO as reductant.

Similar to the supplementation of acetate, the addition of non-physiological substrate propionate during CO cultivation reduced growth lag phase of WT from 5 days to 2 days (Fig. 3A and Fig. S8A), whereas the growth lag phase of the *aor1+2* double KO strain was not altered. An increase in cell density (OD_600_) from 0.61 (no supplementation) to 1.1 (propionate supplementation) and a reduction of headspace pressure to the same level of the WT was observed for the double KO strain (Fig. S8B). The supplementation of propionate increases acetate and ethanol formation, but reduces 2,3-butanediol and lactate production of WT growing on CO (Fig. 3 and Fig. S8). Propionate concentrations remained unchanged and no 1-propanol was detected in cultures of double KO strain (Fig. S8G & H). In contrast, in cultures of the WT strain 24.2 mM propionate was consumed and 20.9 mM 1-propanol produced during the exponential growth phase (Fig. S8G & H).

In the case of butyrate supplementation in the presence of CO, the *aor1+2* double KO strain grew to a similar OD_600_ as the WT and reduced headspace pressure to the same extent (Fig. S9A & B). The KO strain produced 17% more acetate (*p*-value=0.019), 36% more 2,3-butanediol (not statistically significant) and 2.8 mM lactate (the WT produced none) but 44% less ethanol (*p*-value=0.016) than the WT. Consistent with the inability to metabolize acetate and propionate, the KO strain showed no consumption of the supplemented butyrate and produced no 1-butanol (Fig. S9G & H). In contrast, in WT cultures 7.4 mM butyrate was consumed and 6.0 mM 1-butanol produced during the stationary growth phase (Fig. S9G & H).

Taken together, these results demonstrated that the AOR of *C. autoethanogenum* is required for the reduction of carboxylic acids into their corresponding primary alcohols. The apparent wide substrate range of AOR in *C. autoethanogenum* is consistent with the finding that the crystal structure of AOR from *P. furiosus* identified a channel that is sufficiently spacious to accommodate a range of substrates including aliphatic and aromatic aldehydes (Chan et al., 1995). The AOR from *C. autoethanogenum* could therefore be heterologously expressed in butyrate-producing acetogens such as *Clostridium drakei* (Gössner et al., 2008), *Clostridium scatologenes* (Küsel et al., 2000), *Eubacterium limosum* (Genthner et al., 1981) and *Oxobacter pfennigii* (Krumholz and Bryant, 1985) to generate 1-butanol.

### 3.3 Metabolic engineering of direct, AdhE ethanol pathway

AdhE typically consists of an N-terminal acetylating Ald domain followed by a C-terminal Fe-type Adh domain (Extance et al., 2013, Membrillo-Hernandez et al., 2000). Since deletion studies and the characterization of the separate AdhE domains indicate that the Ald and Adh domains are functionally autonomous (Arnau et al., 1998, Chen et al., 2004, Espinosa et al., 2001), the Ald domain and Adh domains of *adhE1* in *C. autoethanogenum* was independently disrupted using ClosTron, generating the strains ‘*adhE1a* KO’ and ‘*adhE1b* KO’, respectively. For the ‘*adhE2’* KO strain, only the Ald domain was targeted. Whilst a number of IFD were also successfully made, the ∆*adhE1* and ∆*adhE1+2* mutants were not explored further as the KO plasmids used to make the deletions could not be cured (Section 3.1). In another mutant, ∆*adhE1*^mut^, an 84 bp region encompassing the *adhE2* promoter was deleted, placing this gene under the control of the stronger *adhE1* promoter (Marcellin et al., 2016, Mock et al., 2015) (Fig. S3).

#### 3.3.1 Heterotrophic growth of *adhE* mutants

**Fig. 5:**
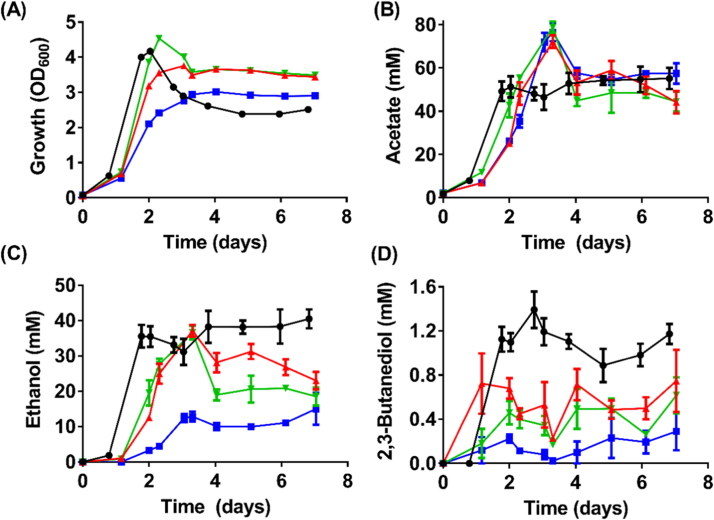
Growth and metabolite profiles of *C. autoethanogenum* WT and *adhE* KO strains on fructose. (A) Growth profile; (B) Acetate profile; (C) Ethanol profile; and (D) 2,3-Butanediol profile. Black circles = WT (n =4); Red triangles = *adhE1a* KO strain (n =3); Green inverted triangles = *adhE1b* KO strain (n =3); Blue squares = *adhE2* KO strain (n =3); Error bars = s.e.m. (For interpretation of the references to color in this figure legend, the reader is referred to the web version of this article.)

Growth of both the *adhE1a* KO and *adhE1b* KO strains on fructose was characterized by a slightly longer lag phase than the WT, but the cells eventually grew to a similar OD_600_ (Fig. 5A). In contrast, the final OD_600_ of the *adhE2* KO strain was 28% lower (*p*-value<0.0001) than the WT (Fig. 5A). Even after 13 days of incubation, 0.92 g/L of fructose was detected in the *adhE2* KO strain culture, whereas all the other strains completely exhausted the substrate prior to day 3 (data not shown). All three *adhE* KO strains reached peak acetate levels of 72.2–76.5 mM, which are 31–43% higher than the WT (*p*-values<0.05) (Fig. 5B). When compared to the WT, both *adhE1* KO strains produced similar amounts of ethanol but the *adhE2* KO strain only generated 37% of the WT ethanol titres (*p*-value=0.0035) (Fig. 5C). All three *adhE* KO strains produced less than half of the 2,3-butanediol recorded in the WT culture (*p*-values<0.05) (Fig. 5D). During growth on fructose, the ∆*adhE1*^mut^ strain produced similar amounts of ethanol as the WT, and qRT-PCR results showed that *adhE2* mRNA levels are higher in the mutant strain (Fig. S10).

The finding that ethanol production from all three *adhE1* inactivation strains (*adhE1a* KO, *adhE1b* KO and ∆*adhE1*^mut^) was not impaired during heterotrophic growth contradicts the finding of Leang et al. (2013), who showed that the deletion of *C. ljungdahlii adhE1* (but not *adhE2*) resulted in a strain that produced 6-fold less ethanol than the WT control. Furthermore, our results in *C. autoethanogenum* demonstrated that *adhE2* inactivation resulted in 63% lower ethanol concentration than the WT.

While *C. autoethanogenum* and *C. ljungdahlii* are very similar on a genetic level (Brown et al., 2014, Marcellin et al., 2016), phenotypic differences that include different ethanol production profiles, are known (Brown et al., 2014, Cotter et al., 2009, Köpke et al., 2010, Liew et al., 2016b, Marcellin et al., 2016, Martin et al., 2016). The AdhE1 and AdhE2 enzymes of *C. autoethanogenum* and *C. ljungdahlii* differ by three and eight amino acids (AA), respectively. It is possible that one of these substitutions results in modification of substrate and cofactor specificities. For example, a single AA change in the Fe-Adh domain of the *C. thermocellum* AdhE changed its cofactor from NADH to NADPH (Brown et al., 2011). One of the *C. autoethanogenum* AdhE2 changes relative to *C. ljungdahlii* resides in the NADH binding site of Adh domain. A change in cofactor specificity would be expected to have significant impact on electron and carbon flows because NADH is commonly used in catabolic reactions whereas NADPH is usually employed as reductant in anabolic processes (Alberts et al., 2002). Another possible explanation for the contradictory phenotypes is that *C. autoethanogenum* may possess other ethanologenic enzymes that compensate for the loss of AdhE activities during fructose growth.

RNA-sequencing experiments in both *C. autoethanogenum* (Marcellin et al., 2016) and *C. ljungdahlii* (Nagarajan et al., 2013, Tan et al., 2013) showed that *adhE1* is transcribed at significantly higher levels when growing on fructose compared to autotrophic growth, which suggests a more important role for this gene under heterotrophic than autotrophic conditions. Unfortunately, the *C. ljungdahlii adhE1* mutant (Leang et al., 2013) has only been characterized during heterotrophic growth (with 5 g/L fructose rather than 10 g/L as in this study) but not for autotrophic growth.

#### 3.3.2 Autotrophic growth of AdhE mutants

**Fig. 6:**
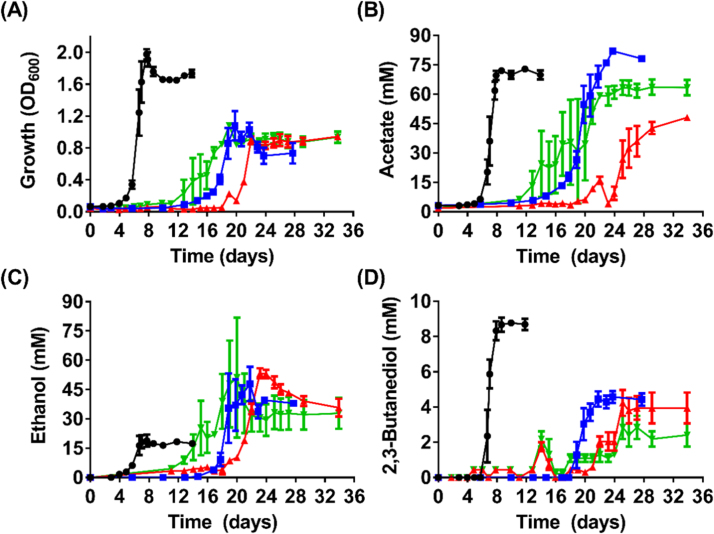
Growth and metabolite profiles of *C. autoethanogenum* WT and *adhE* KO strains on CO. (A) Growth profile; (B) Acetate profile; (C) Ethanol profile; and (D) 2,3-Butanediol profile. Black circles = WT (n =4); Red triangles = *adhE1a* KO strain (n =3); Green inverted triangles = *adhE1b* KO strain (n =2); Blue squares = *adhE2* KO strain (n =3); Error bars = s.e.m. (For interpretation of the references to color in this figure legend, the reader is referred to the web version of this article.)

During growth on pure CO, all three *adhE* KO strains (*adhE1a*, *adhE1b*, and *adhE2*) displayed significant growth deficiencies in the form of prolonged lag phase and 47–55% lower cell density than WT (p-values <0.01) (Fig. 6A), which suggests inefficiency in recycling reducing equivalents. Despite the low biomass, all three *adhE* KO strains consistently generated 154–183% higher titres of ethanol while growing on CO. Specifically, the *adhE1a* KO strain produced 53.4 mM ethanol, 183% more than WT (p-value =0.0005). The *adhE1b* KO strain produced 171% more ethanol (not statistically significant) and the *adhE2* KO strain produced 154% more ethanol than WT (p-value =0.021) (Fig. 6C). The enhanced ethanol production of these *adhE* mutants is also reflected in the peak ethanol/peak acetate ratio of 0.58−1.11, in comparison to 0.26 in WT (Table 1). The substantial improvements in ethanol production were partially offset by a reduction of 48–68% in 2,3-butanediol titres (*p*-values<0.004) (Fig. 6D). Given the similarities in phenotypes between *adhE1a* KO strain and *adhE1b* KO strain, the position of ClosTron insertion within *adhE1* (at Ald domain or Adh domain) played an insignificant role in the overall phenotype of the mutant. The ∆*adhE1*^mut^ strain, which exhibited an upregulated *adhE2* mRNA expression (Fig. S10D), produced 27% more ethanol than the parental strain (not statistically significant; Fig. S11C).

The marked increase in ethanol production exhibited by the *adhE* inactivation strains while growing on CO is in agreement with the hypothesis that the ATP-efficient, indirect ethanol formation route employing AOR is more favourable for autotrophic ethanol biosynthesis (Bertsch and Muller, 2015, Fast and Papoutsakis, 2012, Mock et al., 2015). It has been hypothesized by Mock et al. (2015) that the CoA-linked acetaldehyde dehydrogenase activity measured in the H_2_+CO_2_-grown *C. autoethanogenum* physiologically only facilitate the reuse of the ethanol formed. In the presence of high ethanol concentration and low H_2_ concentration, ethanol oxidation to acetyl-CoA is hypothesized to be coupled to the reduction of 2 CO_2_ to acetate (Mock et al., 2015). In support of this notion, *C. autoethanogenum* WT growing on H_2_+CO_2_ transiently produced 10.3 mM ethanol during exponential growth but thereafter there was a steep decline to 1.8 mM during stationary phase. In addition to the two *adhE* genes, there are 3 other mono-functional *ald* genes (CLAU_1772, 1783 & 3204) in the genome of *C. autoethanogenum*. Accordingly, the generation of a triple *ald* KO strain may further channel carbon and electrons towards acetate synthesis and ethanol formation via AOR.

## 4 Conclusion

Conventional strategies that seek to enhance ethanol production commonly employ the introduction or overexpression of AdhE (Peng et al., 2008, Thapa et al., 2015, Yao and Mikkelsen, 2010). However, given the unique metabolism of acetogens living on the thermodynamic edge of life, we found that the inactivation of *adhE* a better strategy due to diversion of carbon and reducing equivalents towards the ATP-yielding acetate formation (Figs. 4D and 6C). The acetic acid can be reduced to acetaldehyde (via AOR, using reduced ferredoxins) and then ethanol via NAD(P)H-dependent Adh (Fig. 1). Using this strategy we have generated strains that produce up to 180% more ethanol and also accumulate up to 38% less of the undesired acetate by-product. The indirect ethanol pathway has been postulated before (Köpke et al., 2010, Mock et al., 2015) but proof on genetic level was missing. Generation of *aor* mutants confirmed the important role of this route in autotrophic ethanol production and also exhibited that the two isozymes in *C. autoethanogenum* have different roles. To enable this work, we have adapted an allelic exchange strategy that allows generation of stable deletion mutants of multiple genes which has been a limitation in advancing acetogens as platform organisms. Interestingly, lactate but not 2,3-butanediol was found to be the major sink of additional reducing power and further optimization for autotrophic ethanol production could come by combining deletion of lactate dehydrogenase gene *ldh* (Köpke et al., 2014) with deletions of *aor2* and *adhE* genes identified in this study.

## Figures and Tables

**Table 1:** Comparison of peak ethanol to peak acetate or 2,3-butanediol between different *C. autoethanogenum* knockout strains.

**Substrate**	**Strain**	**Peak Ethanol (mM)/Peak Acetate (mM)**	**Peak Ethanol (mM)/Peak 2,3-butanediol (mM)**
**CO**	WT	0.26	2.23
	*aor1* KO	0.12	4.10
	*aor2* KO	1.09	5.90
	*aor1+2* KO	0.22	3.55
	*adhE1a* KO	1.11	12.59
	*adhE1b* KO	0.80	17.94
	*adhE2* KO	0.58	10.47

**H**_**2**_**+ CO**_**2**_	WT	0.16	N/A^a^
	*aor1+2* KO	0.02	N/A^a^

**Fructose**	WT	0.73	29.12
	*aor1* KO	0.41	48.77
	*aor2* KO	1.15	61.51
	*aor1+2* KO	0.42	24.06
	*adhE1a* KO	0.51	49.45
	*adhE1b* KO	0.46	59.00
	*adhE2* KO	0.20	51.79

a N/A=not available because no 2,3-butanediol was detected.
