# Supplementary material for: Metabolic engineering of Clostridium autoethanogenum for selective alcohol production
Source: Metab Eng. 2017 Mar;40:104–14. doi: 10.1016/j.ymben.2017.01.007 (PMC5367853; doi:10.1016/j.ymben.2017.01.007)
Supplement: Supplementary file 1 — Supplementary material [file mmc1.docx]

**Supplementary Information**

**Metabolic Engineering of *Clostridium autoethanogenum* for Selective Alcohol Production**

Fungmin Liew^1,2^, Anne M. Henstra^1^, Michael Kӧpke^2^, Klaus Winzer^1^

Sean D. Simpson^2^, Nigel P. Minton^1,*^

#### *^1^BBSRC/EPSRC Synthetic Biology Research Centre (SBRC), School of Life Sciences, University Park, The University of Nottingham, Nottingham, NG7 2RD, UK*

*^2^LanzaTech Inc., 8045 Lamon Avenue, Suite 400, Skokie, IL, USA*

**Table S1.** Bacterial strains used in this study.

| Strain | Description/Genotype | Source/Reference |
| --- | --- | --- |
| *Escherichia coli* CA434 (HB101) | Conjugative transfer strain. Strain HB101 [*thi-I hsdS20* (r_Ƃ_, m_Ƃ_) *supE44 recAB ara-14 leuB5proA2 lacYl galKI* *rpsL20* (Str^R^) *xyl-5 mt1-1*] carrying the Tra^+^, Mob^+^ plasmid R702 [R702-Tc^R^, Sm^R^, Su^R^, Hg^R^] | M. Young, UCW, Aberystwyth, UK (1) |
| *E. coli* XL1-Blue MRF` Kan | Plasmid storage strain. *∆(mcrA)183 ∆(mcrCB-hsdSMR-mrr)173 endA1 supE44 thi-1 recA1 gyrA96 relA1 lac* [*F’proAB lacI^q^Z∆M15* Tn*10* (Tet^R^)] | Stratagene |
| *Clostridium autoethanogenum* DSM 10061 | Wild-type isolate | DSMZ (German Collection of Microorganisms and Cell Cultures GmbH) |
| *C. autoethanogenum* CauDSM10061-*adhE1a-*115s::CT | Group II intron directionally inserted at upstream Ald domain of *adhE1* (CLAU_3655) gene locus | This study |
| *C. autoethanogenum*  CauDSM10061-*adhE1b-*541s::CT | Group II intron directionally inserted at downstream Adh domain of *adhE1* (CLAU_3655) gene locus | This study |
| *C. autoethanogenum* CauDSM10061-*adhE2-*662s::CT | Group II intron directionally inserted at *adhE2* (CLAU_3656) gene locus | This study |
| *C. autoethanogenum* CauDSM10061-*aor1-*361s::CT | Group II intron directionally inserted at *aor1* (CLAU_0081) gene locus | This study |
| *C. autoethanogenum* CauDSM10061-*aor2-*370s::CT | Group II intron directionally inserted at *aor2* (CLAU_0099) gene locus | This study |
| *C. autoethanogenum* ∆*pyrE* in-frame deletion strain | *pyrE* in-frame deletion created via Allele-Coupled Exchange | This study |
| *C. autoethanogenum* ∆*adhE1* in-frame deletion strain | *adhE1* In-frame deletion in ∆*pyrE* strain | This study |
| *C. autoethanogenum* ∆*adhE1*^mut^ in-frame deletion strain | *adhE1* In-frame deletion with unintended promoter deletion of *adhE2* in ∆*pyrE* strain | This study |
| *C. autoethanogenum* ∆*adhE1+2* in-frame deletion strain | *adhE1* and *adhE2* In-frame deletion in ∆*pyrE* strain | This study |
| *C. autoethanogenum* *aor1*+*2* double KO strain | Group II intron directionally inserted at *aor1* gene locus in ∆*pyrE* strain. Followed by *aor2* in-frame deletion using *pyrE*-mediated allelic exchange. In final step, *pyrE* was restored back to WT | This study |

**Table S2.** Oligonucleotides used in this study

| Primer name | DNA sequence (5` to 3`) | Function(s) |
| --- | --- | --- |
| Univ-0027-F | GCG AGA GTT TGA TCC TGG CTC AG | Amplification and sequencing of 16s rRNA for Eubacteria; (2) |
| Univ-1492-R | CGC GGT TAC CTT GTT ACG ACT T |  |
| P_acsA_-NotI-F | AAGCGGCCGCAGATAGTCATAATAGTTCC | Amplification and cloning of *acsA* (CLAU_1579) promoter region of *C. autoethanogenum* |
| P_acsA-_NdeI-R | TTCCATATGAATAATTCCCTCCTTAAAGC |  |
| aor1-NdeI-F | AATTCATATGTATGGTTATGATGGTAAAGTATTAAG | SOE PCR to mutate two internal *Nde*I restriction sites and clone *aor1* (CLAU_0081) from *C. autoethanogenum* |
| aor1-SOE-B1 | CTAAATCATAAGAACCACAGTCAGATCC |  |
| aor1-SOE-C1 | CTGACTGTGGTTCTTATGATTTAGATGC |  |
| aor1-SOE-C2B | ATTCCAGCTTATGATCCAAGGGG |  |
| aor1-SOE-B2B | CCCCTTGGATCATAAGCTGGAAT |  |
| aor1-KpnI-R | CTAGGTACCCGAATCAAACTAGAACTTACC |  |
| adhE1a-115s-F | ACTGCTAAAAATTAGGAAAGAGGTGTCGCT | PCR screening for integration of Intron I into upstream Ald domain of *C. autoethanogenum* *adhE1* |
| adhE1a-115s-R | CTGCATCTCTCTCTAAAACTCCACAGGT |  |
| adhE1b-541s-F | ATTTGCAGTAATCACTGATGAAAAAACAGGAGC | PCR screening for integration of Intron I into downstream Adh domain of *C. autoethanogenum adhE1* |
| adhE1b-541s-R | GTAAATATTCAAATATCAACTTTACTGCTTCAAGGGC |  |
| adhE2-662s-F | GCTAAGATAGTACTTGATGCAGCAGTTAAAGC | PCR screening for integration of Intron I into *C. autoethanogenum adhE2* |
| adhE2-662s-R | CTGACTGCTCTGAAGCACAAATCATACCA |  |
| aor1-361s-F | GCACCGCTTACAGGAACTATAGG | PCR screening for integration of Intron I into *C. autoethanogenum* *aor1* |
| aor1-361s-R | CAGCTAAAGCTATTTTTCCAGTTCC |  |
| aor2-370s-F | GCACCTGTTCCAACAAGCGGAA | PCR screening for integration of Intron I into *C. autoethanogenum* *aor2* |
| aor2-370s-R | ACTTTTCCCCAGCTGGTCCTATGC |  |
| adhE1-IFD-F | CTAACATCAAGGGGTTTATTTGTCAC | Screening of double crossover and subsequent sequencing of *C. autoethanogenum* *adhE1* |
| adhE1-IFD-R | GTTACCTTAGTTACCTTCATTAATGACAC |  |
| adhE1-seq-F | GCAAGATTTCAATAAAGGGTGTATTTTACC | Screening of double crossover and subsequent sequencing for in-frame deletion of *C. autoethanogenum* *adhE1* and *adhE2* |
| adhE2-seq-R | CAATATATTATAAAATAGGGAGTGGAATGTTCC |  |
| aor2-seq-F | CACCTTTAAGAGATAGAAAAGAAGATATCCC | Screening of double crossover and subsequent sequencing of *C. autoethanogenum aor2* in-frame deletion |
| aor2-seq-R | TTTTCCTAATGCTTTTACCTCTATTAGTGG |  |
| ACE-plasmid-F | TGAAGTACATCACCGACGAGC | Anneal to region prior to left homology arm (LHA) of ACE plasmid. To be used for screening of single crossover mutant at LHA |
| ACE-plasmid-R | TTCCCAAATCCTTACATCTCCCC | Anneal to region prior to right homology arm (RHA) of ACE plasmid. To be used for screening of single crossover mutant at RHA |
| adhE1-ald-LHA-SacII-F | TATCTTCCGCGGGTATTAAATTGTAAAATATCC | SOE-PCR to construct left homology arm for in-frame deletion of *C. autoethanogenum adhE1* or *adhE1 & 2* |
| adhE1-IFD-LHA-R | ATTTAATAATTACATTAATTAGCGACACCTCTTTCC | SOE-PCR to construct homology arms for in-frame deletion of *C. autoethanogenum adhE1* |
| adhE1-IFD-RHA-F | GGTGTCGCTAATTAATGTAATTATTAAATAAAAATGGTG |  |
| adhE1-IFD-RHA-AscI-R | TTGGCGCGCCTAGTGATTTAAATATTGC |  |
| adhE1&2-SOE-B | ATTATATATTTACATTAATTAGCGACACCTCTTTCC | SOE-PCR to construct homology arms for in-frame deletion of *C. autoethanogenum adhE1 & 2* |
| adhE1&2-SOE-C | CGCTAATTAATGTAAATATATAATAAATTGAATATAGTAAAC |  |
| adhE1&2-RHA-AscI-R | GGTGGCGCGCCTTTATTTCC |  |
| aor2-LHA-SacII-F | ACTCCGCGGCTAAAGTAAGTAAG | SOE-PCR to construct homology arms for in-frame deletion of *C. autoethanogenum aor2* |
| aor2-LHA-R | CTCTAATTAATCAAACTACATAAAAACCCTCC |  |
| aor2-RHA-F | GGAGGGTTTTTATGTAGTTTGATTAATTAGAG |  |
| aor2-RHA-AscI-R | TTTGGCGCGCCTATAGTATCTG |  |

Notes: Restriction sites are underlined.

**Table S3.** Plasmids used in this study

| Plasmid | Description | Source/Reference |
| --- | --- | --- |
| pMTL83151 | *Escherichia coli*/*Clostridium* modular shuttle vector, pCB102, ColE1+tra, Cm^R^/Tm^R^ | Heap et al., 2009 (3) |
| pMTL83151-P_acsA_ | Overexpression plasmid with *C. autoethanogenum acsA* (CLAU_1579) promoter cloned between *Not*I and *Nde*I sites | This work |
| pMTL83151-P_acsA_-aor1 | Overexpression plasmid of *C. autoethanogenum aor1* | This work |
| pMTL84151 | *E. coli*/*Clostridium* modular shuttle vector, pCD6, ColE1+tra, Cm^R^/Tm^R^ | Heap et al., 2009 (3) |
| pMTL-AMH101 | ACE plasmid for creation of the ∆*pyrE* (CLAU_1436) in-frame deletion strain in *C. autoethanogenum.* Plasmid contains functional *C. acetobutylicum pyrE* (CLAU_1436) as counter selectable marker | This work |
| pMTL-AMH102 | ACE plasmid for repair of the ∆*pyrE* (CLAU_1436) in-frame deletion strain in *C. autoethanogenum* | This work |
| pMTL84151-∆adhE1 | ACE plasmid for the creation of *adhE1* in-frame deletion in *C. autoethanogenum.* Plasmid contains a functional *C. acetobutylicum pyrE* as counter selectable marker | This work |
| pMTL84151-∆adhE1+2 | ACE plasmid for the creation of *adhE1* and *adhE2* in-frame deletion in *C. autoethanogenum*. Plasmid contains a functional *C. acetobutylicum pyrE* as counter selectable marker | This work |
| pMTL84151-∆aor2 | ACE plasmid for the creation of *aor2* (CLAU_0099) in-frame deletion in *C. autoethanogenum*. Plasmid contains functional *C. acetobutylicum pyrE* | This work |
| pMTL007C-E2 | Clostridial expression vector for the ClosTron, containing a directed Group II intron with Erm RAM, flanked by FRT sites, ColE1, pCB102, Cm^R^/Tm^R^ | Heap *et al*. (2007) (4) |
| pMTL007C-E2::adhE1a-115s | ClosTron vector targeting Group II insertional knockout at the *C. autoethanogenum* *adhE1* locus (upstream Ald domain of CLAU_3655) | This work |
| pMTL007C-E2::adhE1b-541s | ClosTron vector targeting Group II insertional knockout at the *C. autoethanogenum adhE1* locus (downstream Adh domain of CLAU_3655) | This work |
| pMTL007C-E2::adhE2-662s | ClosTron vector targeting Group II insertional knockout at the *C. autoethanogenum adhE2* locus (CLAU_3656) | This work |
| pMTL007C-E2::aor1-361s | ClosTron vector targeting Group II insertional knockout at the *C. autoethanogenum aor1* locus (CLAU_0081) | This work |
| pMTL007C-E2::aor2-370s | ClosTron vector targeting Group II insertional knockout at the *C. autoethanogenum aor2* locus (CLAU_0099) | This work |

**Table S4.** Primers and TaqMan MGB probes used in this study.

| Assay ID | Assay Name | Forward Primer Sequence | Reverse Primer Sequence | Reporter 1 Sequence |
| --- | --- | --- | --- | --- |
| AI89K3D | *gyrA* | GAATTGATGGAAACAATAAAACATCTCAAGGA | AGTTTTTCTTTCATCACCATATTTATCTCTTATCTCAATTA | TCAGCAGCAATTCTTT |
| AIAAZ86 | *rho* | GGCATTATTGTATGTTGAAAAAGTAAATGGAGA | ACAGGAGTGAGTGTTTCAAAAGGTT | TCCCACTGCCCTTTCT |
| AII1NGQ | *adhE2* | CCTGAAGAAGAGCCCTTTGCT | AGTGCTTCATCAAAATCTTTTGCTTTGT | CTGGAGACAGCTTTTC |


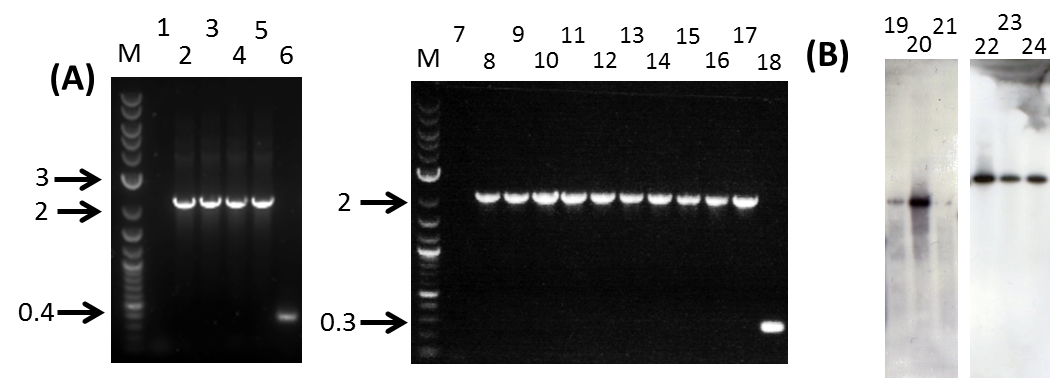


**Figure S1.** Screening and validation of *aor1* and *aor2* KO strains. (A) Gel electrophoresis of PCR using exon-spanning primers; Lanes 2 - 5 = *aor1* KO strains; Lanes 8 – 17 = *aor2* KO strains; Lanes 1 & 7= Non-template controls; Lanes 6 & 18 = WT controls; M = NEB 2-log DNA ladder in kb; (B) Southern Blot analysis of *Hin*dIII digested genomic DNA of *aor1* KO strains (lanes 19 – 21), and *aor2* KO strains (lanes 22 – 24). Arrows and the accompanying numbers denote the fragment sizes of DNA ladder in kilobases.


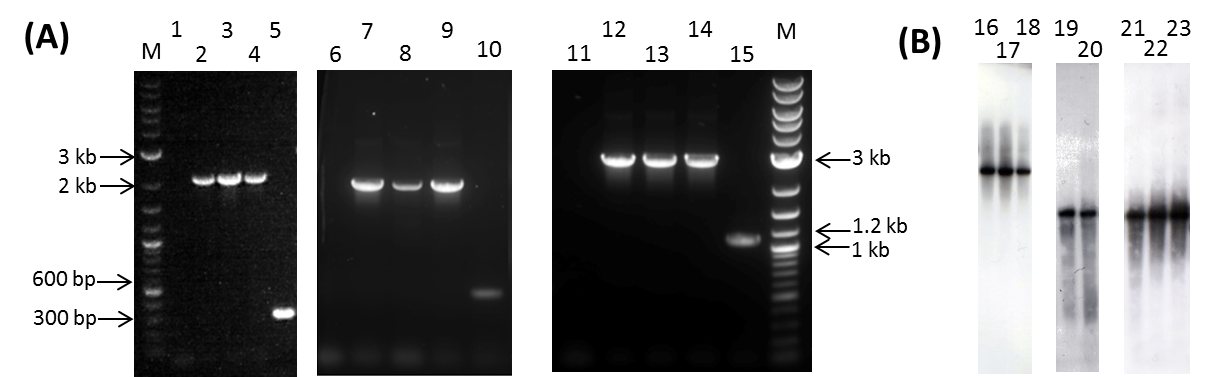


Figure S2. Screening and validation of *adhE1a*, *adhE1b*, and *adhE2* KO strains. (A) Gel electrophoresis of PCR using exon-spanning primers; Lanes 2 – 4 = *adhE1a* KO strains; Lanes 7 – 9 = *adhE1b* KO strains; Lanes 12 – 14 = *adhE2* KO strains; Lanes 1, 6, & 11 = Non-template controls; Lanes 5, 10 & 15 = WT controls; M = NEB 2-log DNA ladder; (B) Southern Blot analysis of *Hin*dIII digested genomic DNA of *adhE1a* KO strains (lanes 16 – 18), *adhE1b* KO strains (lanes 19 & 20), and *adhE2* KO strains (lanes 21 – 23).


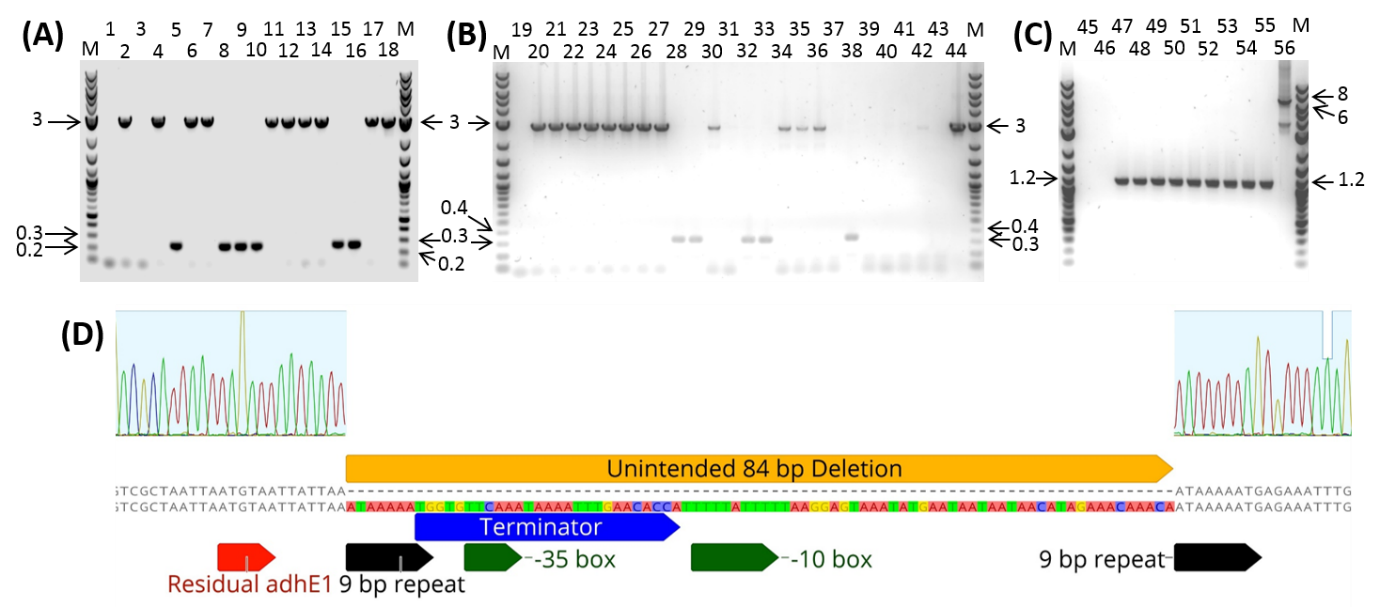


**Figure S3.** Screening of *C. autoethanogenum* ∆*adhE1*^mut^, ∆*adhE1* and ∆*adhE1+2* strains. (A) PCR screening of ∆*adhE1*^mut^ strains; (B) PCR screening of ∆*adhE1* strains; (C) PCR screening of ∆*adhE1+2* strains; and (D) Nucleotide alignment of Sanger sequencing read from ∆*adhE1*^mut^ strain highlighting the unintended 84 bp deletion in the inter-genic region of *adhE1* and *adhE2*. Green peak = thymine; Red peak = adenine; Brown peak = guanine; Blue peak = cytosine; Lanes 5, 8, 9, 10, 15 & 16 = ∆*adhE1*^mut^ strains; Lanes 28, 29, 32, 33 & 38 = ∆*adhE1* strains; Lanes 47 – 55 = ∆*adhE1+2* strains; Lanes 2, 4, 6, 7, 11, 12, 13, 14, 17, 20, 21, 22, 23, 24, 25, 26, 27, 30, 34, 35, 36 & 42 = WT revertants; Lanes 1, 19 & 45 = non-template controls; Lanes 18, 44 & 56 = WT genomic DNA control; No PCR product was detected in lanes 3, 37, 39, 40, 41, 43 & 46; M = NEB 2-log DNA ladder. Arrows and the accompanying numbers denote the fragment sizes of DNA ladder in kilobases.


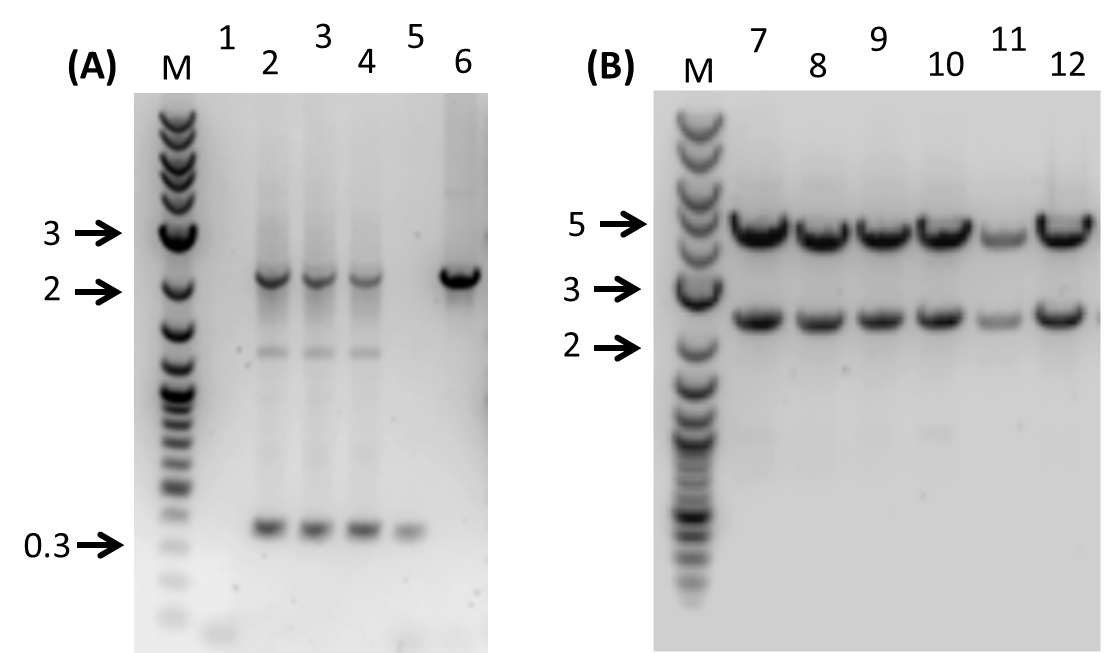


**Figure S4.** Verification of complemented *aor1* strain. (A) PCR of genomic DNA from *aor1* complemented *aor1* strain (lanes 2-4) using exon spanning *aor1* primers; M = NEB 2-Log DNA ladder; 1 = non-template control; 5 = WT genomic DNA control; 6 = *aor1* KO control; (B) *Asc*I and *Pme*I restriction digests of rescued plasmids pMTL83151-P_acsA_-aor1 from complemented strain (lanes 7-12). Arrows and the accompanying numbers denote the fragment sizes of DNA ladder in kilobases.


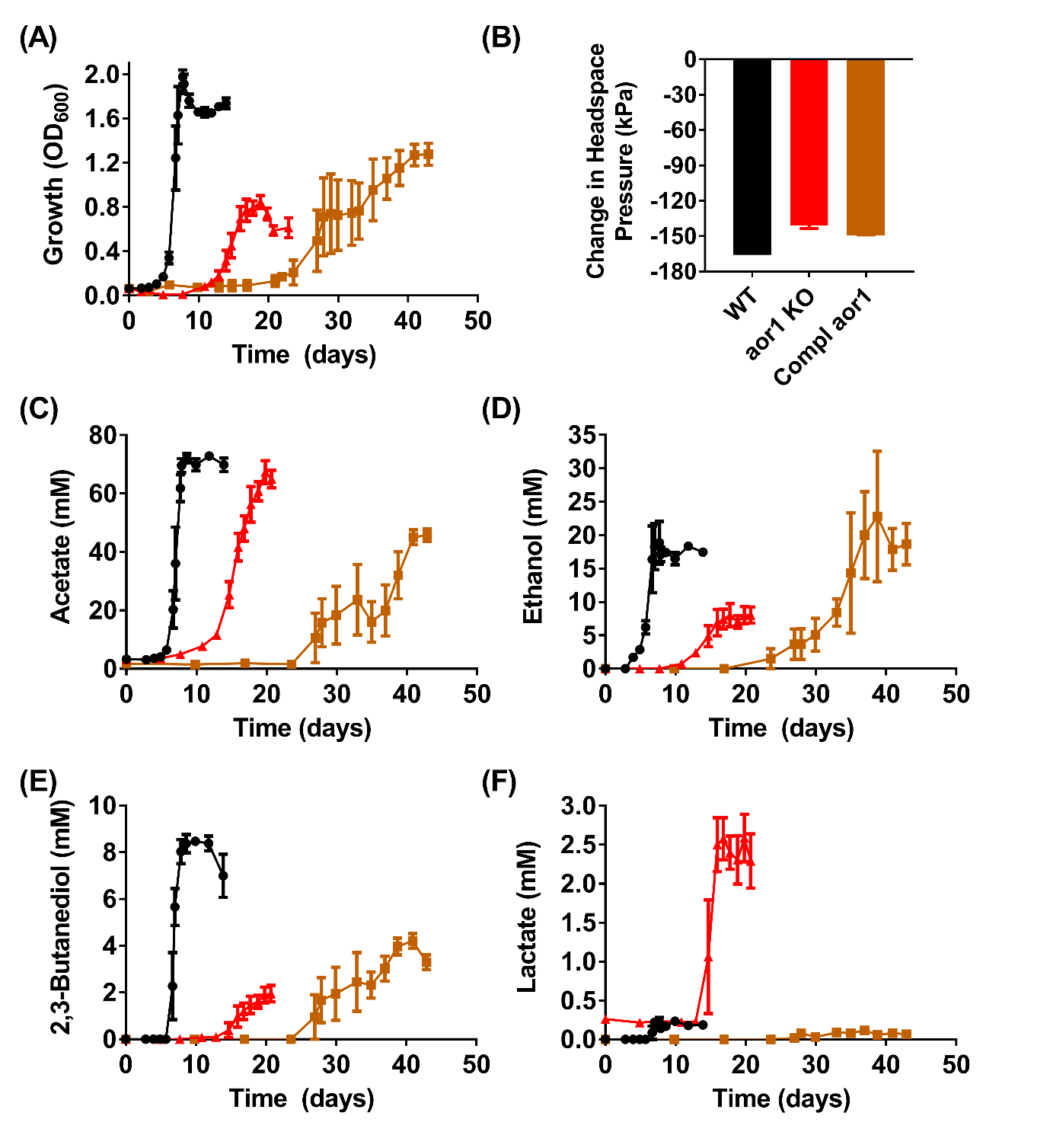


**Figure S5.** Growth, headspace pressure and metabolite profiles of *C. autoethanogenum* WT, *aor1* KO, and complemented *aor1* strains on 200 kPa CO. (A) Growth profile; (B) Change in headspace pressure (C) Acetate profile; (D) Ethanol profile; (E) 2,3-Butanediol profile; and (F) Lactate profile. Black circles = WT (n = 4); Red triangles = *aor1* strains (n = 4); Brown squares = complemented *aor1* strains (n = 3); Error bars = s.e.m.


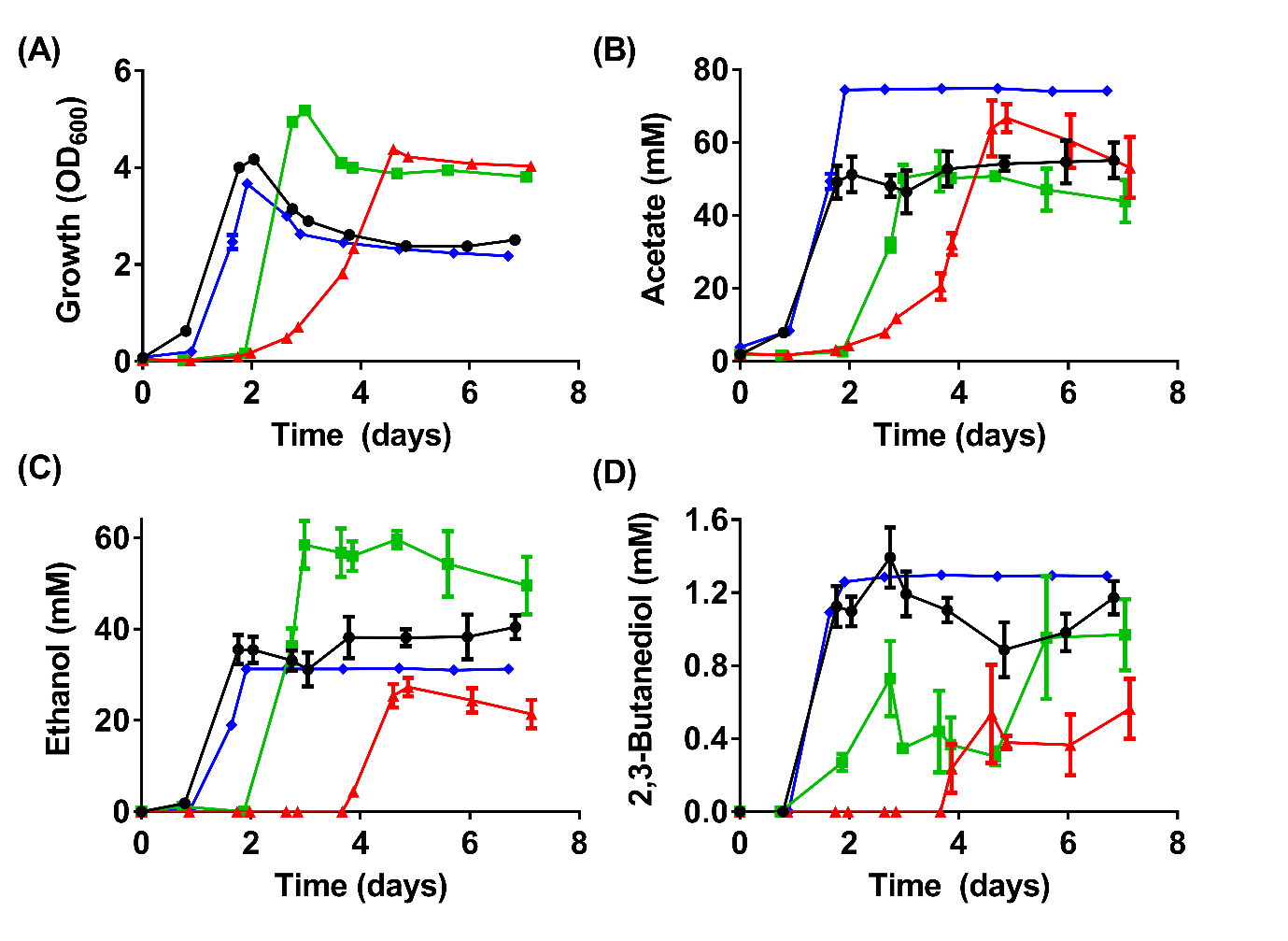


**Figure S6.** Growth and metabolite profiles of *C. autoethanogenum* WT, *aor1* KO, *aor2* KO and *aor1+2* KO strains on fructose. (A) Growth profile; (B) Acetate profile; (C) Ethanol profile; and (D) 2,3-Butanediol profile. Black circles = WT (n = 4); Red triangles = *aor1* strain (n = 3); Green squares = *aor2* strain (n = 3); Blue diamonds = *aor1+2* KO strain (n = 4); Error bars = s.e.m.


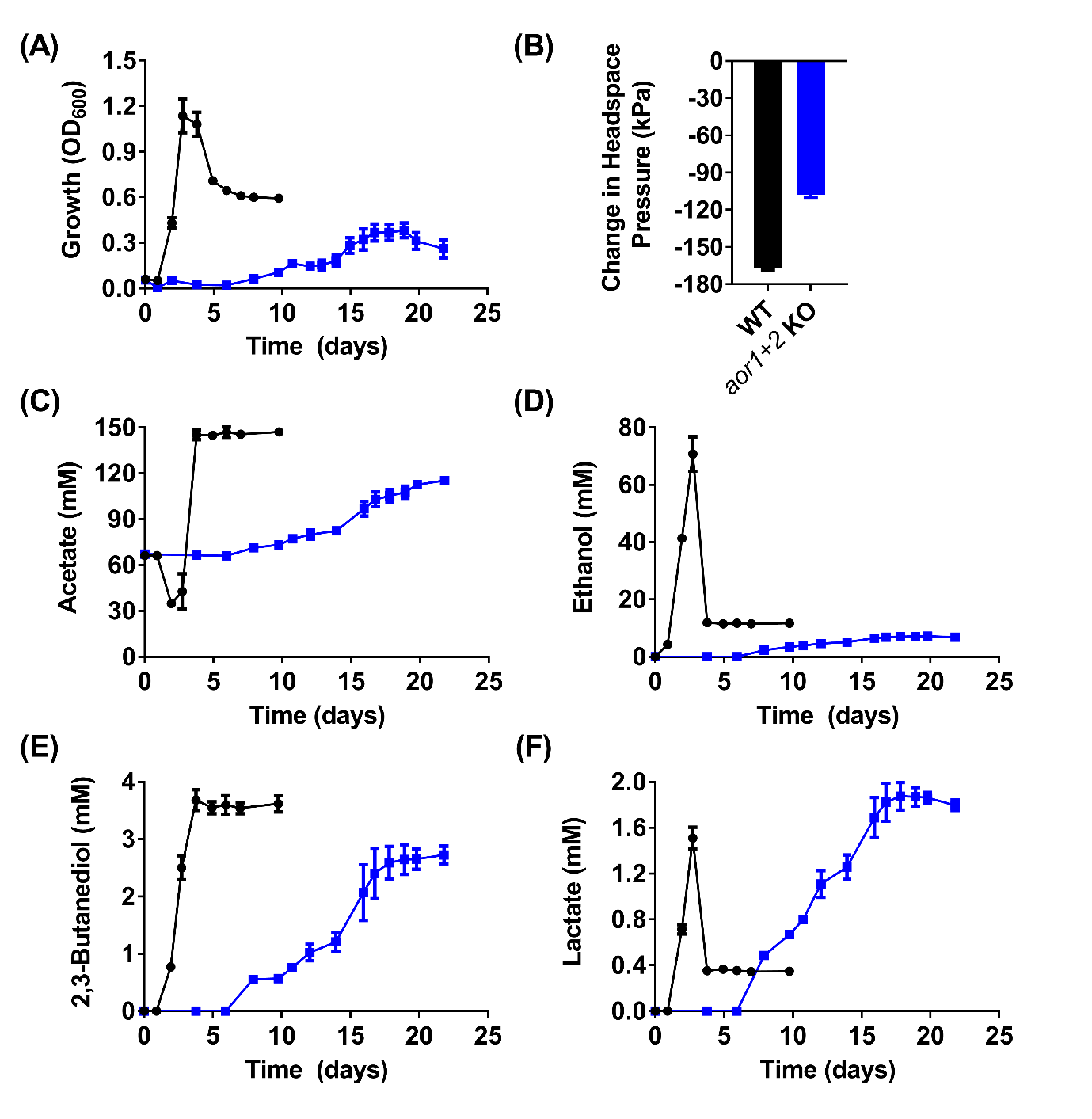


**Figure S7.** Growth, headspace pressure and metabolite profiles of *C. autoethanogenum* WT and *aor1+2* KO strain in the presence of 60 mM acetate and 200 kPa CO. (A) Growth profile; (B) Change in headspace pressure; (C) Acetate profile; (D) Ethanol profile; (E) 2,3-Butanediol profile; and (F) Lactate profile. Black circles = WT; Blue squares = *aor1+2* KO strain; n = 3; Error bars = s.e.m.


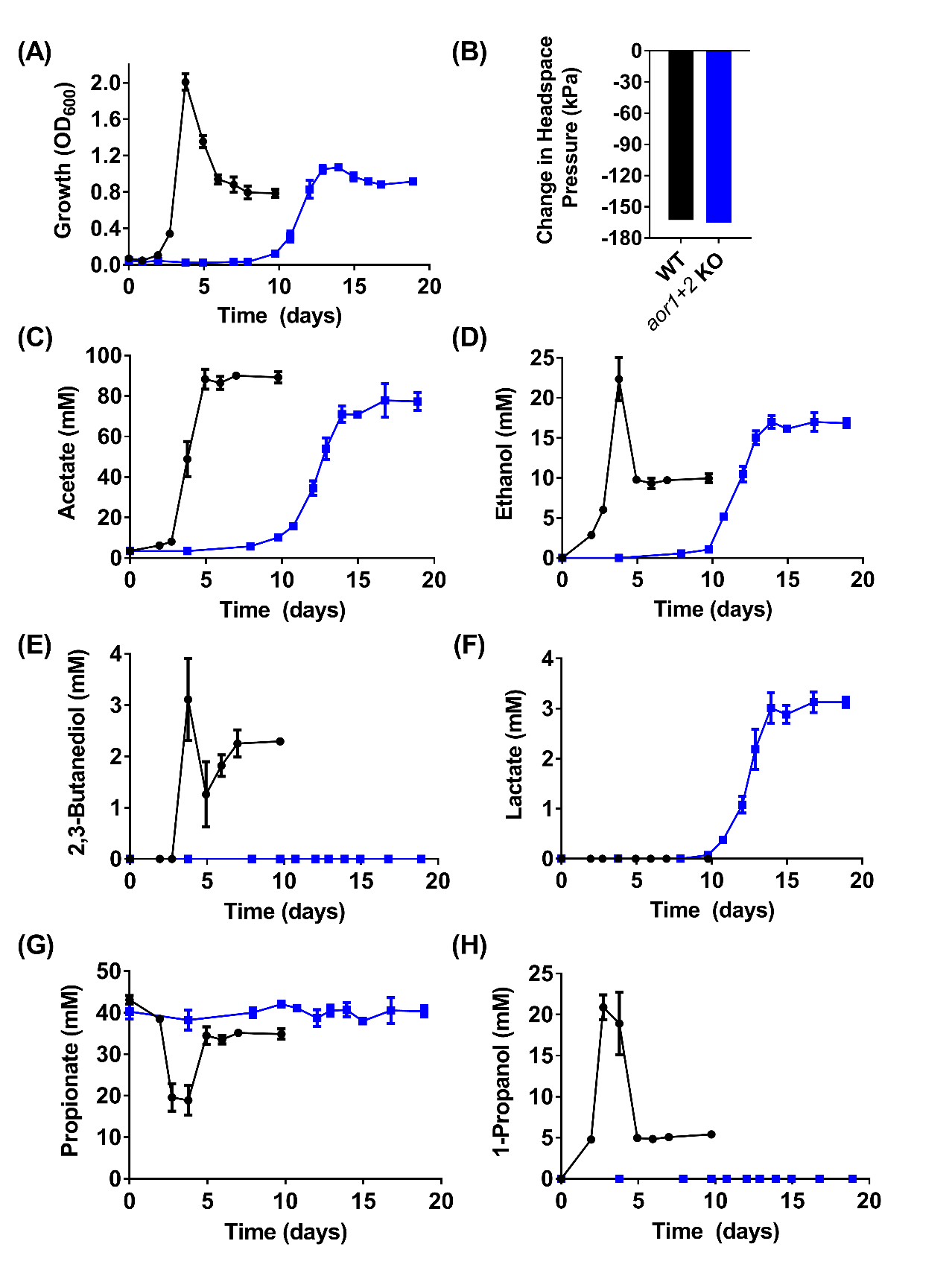


**Figure S8.** Growth, headspace pressure and metabolite profiles of *C. autoethanogenum* WT and *aor1+2* KO strain in the presence of 40 mM propionate and 200 kPa CO. (A) Growth profile; (B) Change in headspace pressure; (C) Acetate profile; (D) Ethanol profile; (E) 2,3-Butanediol profile; (F) Lactate profile; (G) Propionate profile; and (H) 1-Propanol profile. Black circles = WT; Blue squares = *aor1+2* KO strain; n = 3; Error bars = s.e.m.


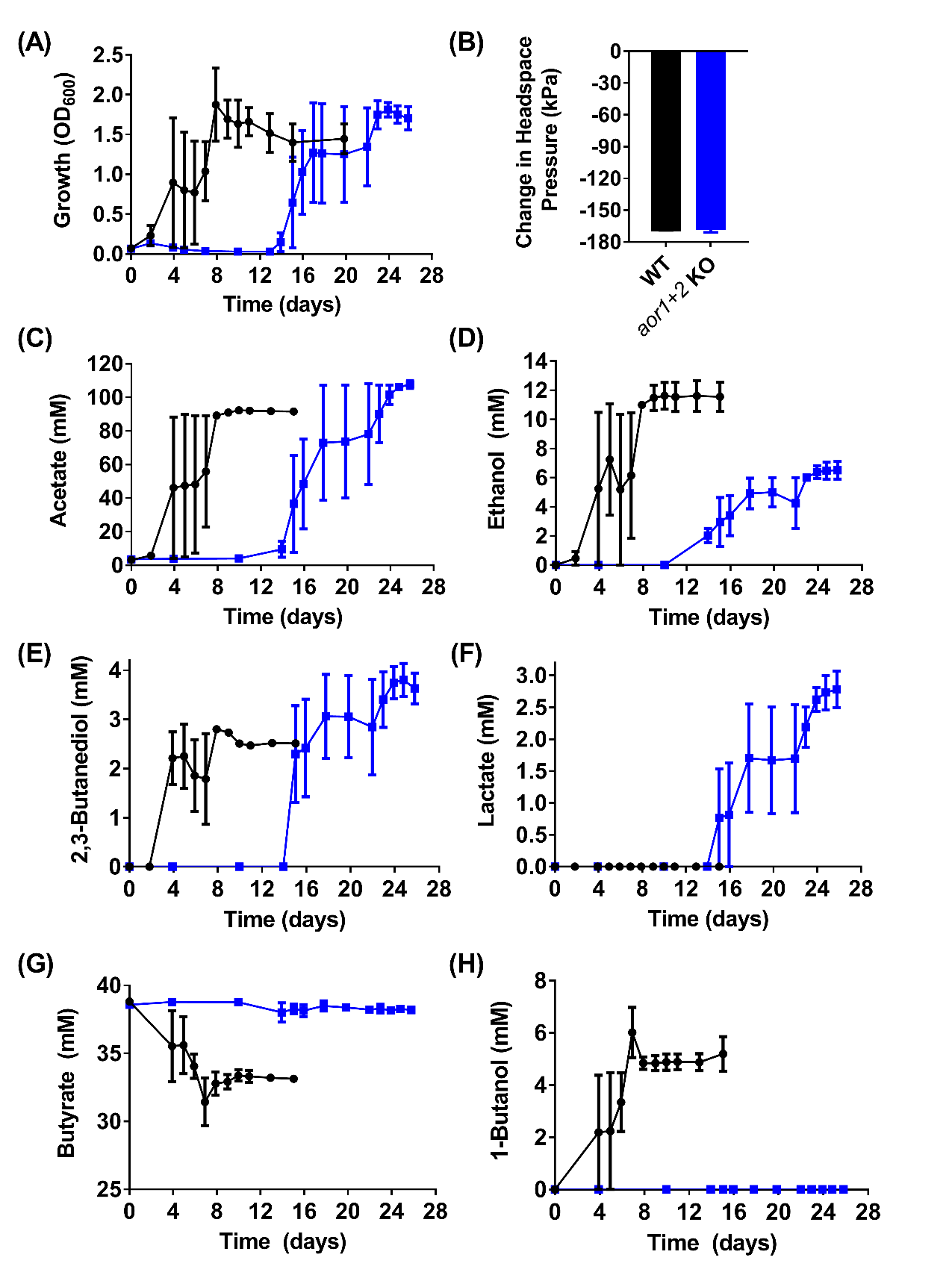


**Figure S9.** Growth, headspace pressure and metabolite profiles of *C. autoethanogenum* WT and *aor1+2* KO strain in the presence of 40 mM butyrate and 200 kPa CO. (A) Growth profile; (B) Change in headspace pressure; (C) Acetate profile; (D) Ethanol profile; (E) 2,3-Butanediol profile; (F) Lactate profile; (G) Butyrate profile; and (H) 1-Butanol profile. Black circles = WT; Blue squares = *aor1+2* KO strain; n = 3; Error bars = s.e.m.


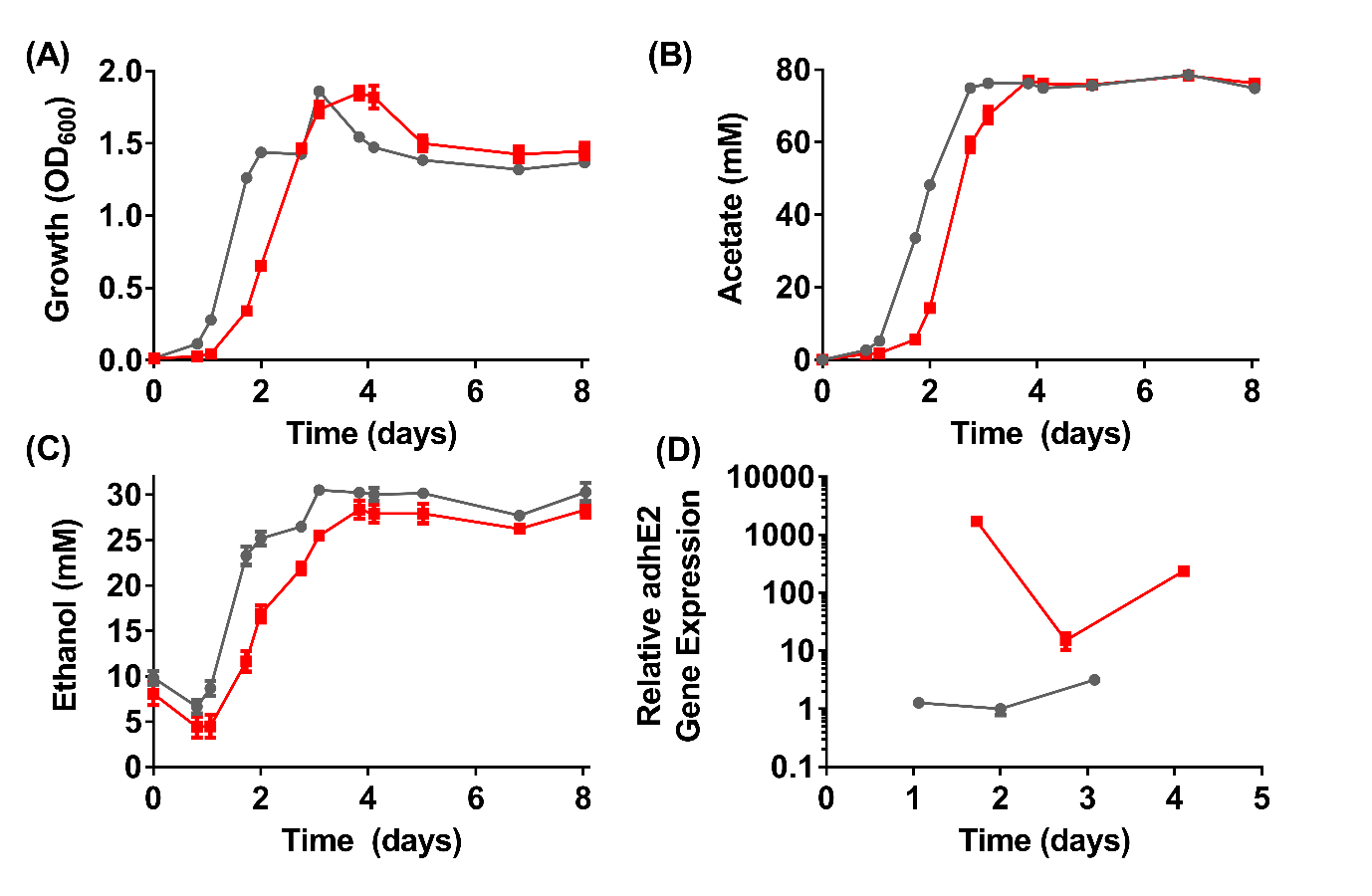


**Figure S10.** Growth, metabolite and *adhE2* transcript profiles of *C. autoethanogenum* ∆*pyrE* and ∆*adhE1*^mut^ strains on fructose. (A) Growth profile; (B) Acetate profile; (C) Ethanol profile; and (D) Relative *adhE2* mRNA profile. Grey circles = ∆*pyrE* (n = 3); Red squares = ∆*adhE1*^mut^ (n = 3). Error bars = s.e.m.


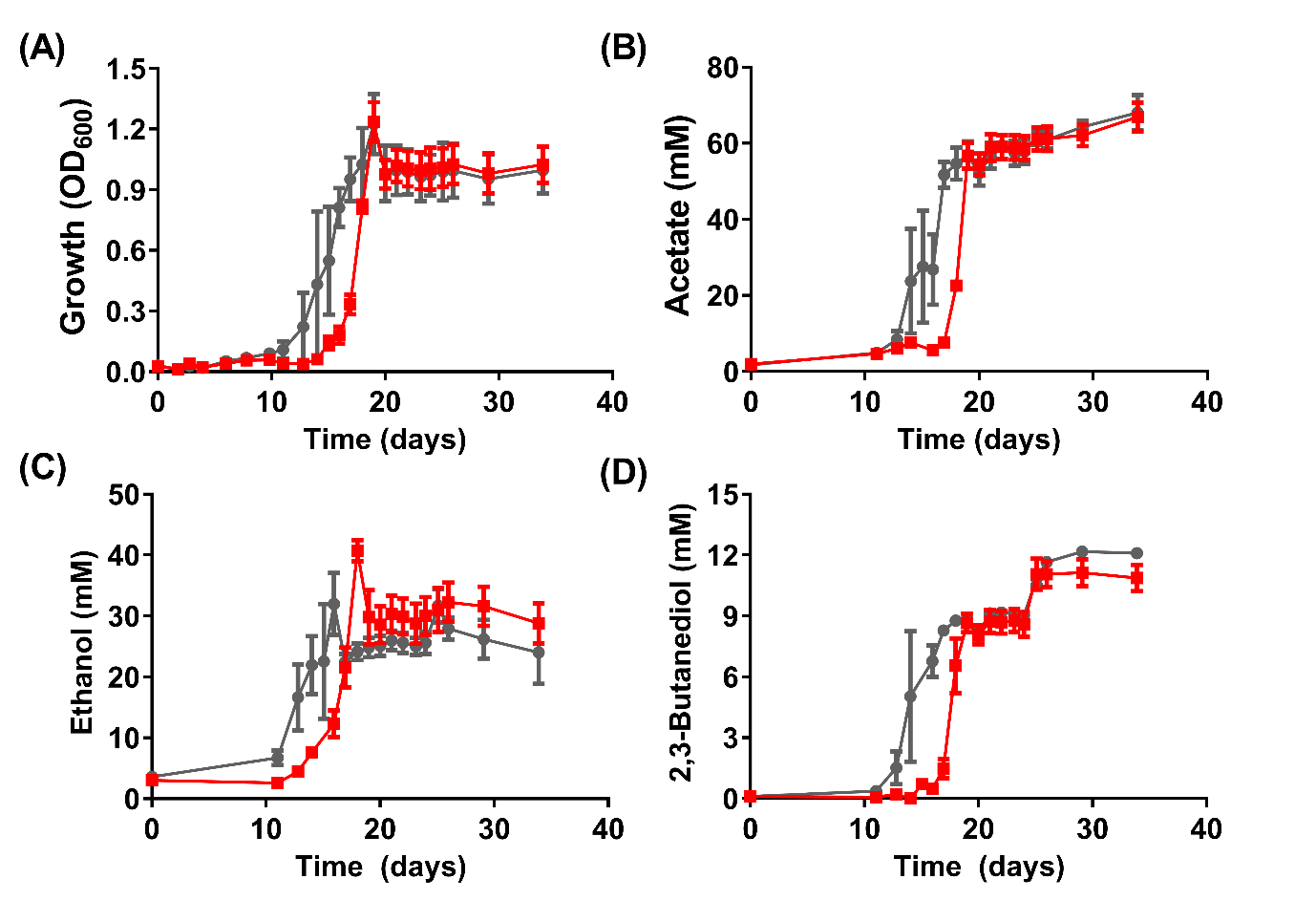


**Figure S11.** Growth and metabolite profiles of *C. autoethanogenum* ∆*pyrE* and ∆*adhE1*^mut^ strains on 200 kPa CO. (A) Growth profile; (B) Acetate profile; (C) Ethanol profile; and (D) 2,3-butanediol profile. Grey circles = ∆*pyrE* (n = 3); Red squares = ∆*adhE1*^mut^ (n = 3). Error bars = s.e.m.

**Supplementary nucleotide sequence 1 (plasmid pMTL-AMH101)**

**pyrE* left homology arm is shown in blue

**pyrE* right homology arm is shown in purple

**pyrE* from *C. acetobutylicum* is shown in red

**catP* selectable marker is shown in Green

GGATAAAAAAATTGTAGATAAATTTTATAAAATAGTTTTATCTACAATTTTTTTATCAGGAAACAGCTATGACCGCGGAAGGGCATTTTCTACTTTCTTCAGGAAAACACAGTGATAGGTATTGTCAGTGTGCAAAACTTTTACAGTATCCTGACAGGGCAAAAGATGTAATAGCAGTTATTGCAGACAAATTAGAGAATGTTGACTATGATAAAATAGTTGGACCTGCAATGGGGGGGATATTAGTTTCCTATGAACTTGCAAGGCAAACGGGCAAACCAGGAATATTTGCTGAAAGGCAAAATGGAAATATGACTATAAGAAGGGGATTTGAAATAAAAGAAGGAGAAAAAATTATAATTTCTGAAGATGTGGCGGCCGCTGTATCCATATGACCATGATTACGAATTCGAGCTCGGTACCCGGGGATCCTCTAGAGTCGACGTCACGCGTCCATGGAGATCTCGAGGCCTGCAGACATGCAAGCTTGGCACTGGCCGTCGTTTTACAACGTCGTGACTGGGAAAACCCTGGCGTTACCCAACTTAATCGCCTTGCAGCACATCCCCCTTTCGCCAGCTGGCGTAATAGCGAAGAGGCCCGCACCGATCGCCCTTCCCAACAGTTGCGCAGCCTGAATGGCGAATGGCGCTAGCGTCGAAAAAATCAATGCACGATGCAGAATTGACAATTAAATTATTAGATTTTAGATTTTTCATAACGCTCACGGAGAAAAATCCACAGAAATTGTGAATTGATTACTTAAAAATAAGCCTATTTCTATAGCACATTAACTGTGTTTTTAGGAATAGGCTTATTTAAGTAAATAGCTTAAATAAGCCTTTCACAATTTTATGGAGTTTTACAAAAAATTATTACTATTTATAAGATTTTTGAGGTATAATAAGTAATGAATTCCTACAATATTATTTATTATATACATGAAAAATTAATAGTGATTTTTTATTGTGTATAATTTTATATAATGGATAATCCTTTGGGAATATTAATCAGGGAATCACCACTTTCTATAATGGGTGATTTAACTTCCCATAGTTATTAATACTAAGTATAGGAGGAGTTTTTTAGTGGGTAAACCAAGTATTTTTAGCAAAGAATATGAAAAGAAAATGCAGCGGCATAAGAGAAATAAAGTTATACTTATTGTAGTATGTTTGATGGCAGCAGTGTTAGTTGTAGTTTACATAAGGGGAGCATTTAGAGATGTAGTAAAGGAAACAAGTAAAGTAAAGAATAATATTGTTTCTGAAAACAAACAGACAAAAAATAATACGGATAGTCAAAAATCATCTCAACCAAATACTCAAACTAAGTCTAAGGATGCAGCAAGTCAGAATTCATATAAAATAAAATTGAGTAGTGGAAAAGATGTAAGTTTAATATATCAAGGTAAGGGTAATGATAAAGTATTTAAGGCAGATAGCAGCGGTACATATGATGTAAGCCCATCAGGTAAGAATGCACTTTTGTTTGATGATAAGTCTCAGAGTATATTGTTAGTGGATGCAAGTGGAAATAAGCAGGATATAACAAATCCACAGTATGTATCTACTACAGGAACTGTAATAGCAAAAGATTCTCAATTAACTAATAATCCAGGATATGTATGGTGTTCTTCACCTAAATTTATAGATGACAATAATATTGCATATGTAAGTCAACTCCCATGGATTGGAAAAACTACAAAATACGTGTGGATAGAAAGCTTGCAGAATAAAAATCATGTTATGGTTCAGAATATAGAAGGTGAAGATATAAAGTTTCAGGGAATAAATGAAAAGGGACTTACGGCCATTGTAGATGGTAAAACTGTTTATTTAACGGCATCAGGTTCCGTTACCCAGTAGGCGCGCCCGCCCTTAAGTCTAAAAATTAGGGGAGATGTAAGGATTTGGGAAAAATAGAAGATGTTATAATCATAAATATGGTATTCGTAGGCTTAAAGTCAAAAAGGAGGTGAAATATAAATAGATTTTTAGCTAAATTAAGTAAGAAATAGGAGGAGATTTATTGAACAAAAAATTAGAAAAACCATTTGTATATAAGAGAGAGTACGATTTGACTGGATATGATGTTGAAATTTTACAAAAATATGAGTTAGAACAAGCAATATATGTTTATGTTGGGAGTAGTTGTGCATATAACATGAGAGCTAGAAGTAGTAAATGGAGATACCATATAAGAACAAATAATAAGTCTATATGTTGTAACATTAAAAATTTTATACATAACTTGGAATTGTTTTATAAAATGGAATTAAAGTTGTCAGATAATATTATTAATGATAAGCTATACTATAGCAATATAGCAGAGTTTGAAGAATTTGAAACACTAGAAAAAGCTAGAGAGGTAGAAAGTACTATTATAAGTCAATATCAATTTTTAGATTCTATAAATCACATGTTAAAACAAAAAATAATTTTATTGAGTAATAAGGATAGTGTGTTAAACATAACTAAAAATGGAAATACAAATTATTTGAAAGTAAAAAATAAATACATAGAAAAACATAAGAACAAGCCAATAATGAGATACCATATCAACTGTCAATTCAATACAGATGGAAGTGTCAAAAGTATTACACAGGAGTTTGAACCAATATTGGAATTAAACAAAAAAAATACCCTAAGCCGACCAAGCAGAGTATTTTTAAAATAATATTTTAAGATAACAACAAAATGAGATAATACTACTAGACAATGACAACTCAACTACCAATTGAGTTTATGGAGCTACCAACTCCAATATCGGTCTAACTGATTAAGTATCTGTAGTTATATAATAATATTGCTATCAATTTTAGCATCTTAACAATATTATTATACATACTAAGCTAAAATTATTCAATAGTTGTAAAAGTTGATTAGTCAATAAGTATATATTTAATGTAGTGTTATCTCTTAAAAAAACTAGATAAGGAGATAATAAATATATGGAACAATTAGATTCAAAATATAAGTTGAAAAAATTTCTAATGGCAGTATTTAGAGATGGTATAGGACAAGGAAATAATCTTATTGATAATGAATATGTTAGAGTATTTCAAAATAATAAAAGTAATAGTAAACAATTAGAACTCGGAGAAGAATTTAAAGAATATAGTAAAACAACTTTTTTTAAAAATATAGATGATATAGTAGAATTTACCTTCGCAAAAAATATTTATTATGAAAATACATTTTTTAACCTATGTACTACTGATGGAAAAGCAGGAACCAATGAAAACTTAATAAATAGATATGCATTAGGATTTGATTTTGACAAAAAAGAATTAGGACAAGGTTTTAATTATAAAGATATAATTAATTTATTTACTAAGATAGGATTACATTATCATATCCTAGTTGATAGTGGAAATGGATTCCATGTTTATGTGCTAATTAATAAAACTAATAACATTAAGTTAGTATCAGAAGTTACAAATACATTAATAAATAAATTGGGTGCAGATAAACAAGCAAATTTATCTACTCAAGTATTAAGAGTACCTTATACATATAATATTAAAAATACTACTAAACAAGTAAAAATAATACACCAAGACAAAAATATATATAGATATGACATAGAAAAGTTAGCTAAAAAATATTGCAAAGATGTAAAAACAGTAGGTAATACTAATACAAAATATATATTAGATAGTAAGCTACCAAATTGTATAGTAGATATTTTAAAAAATGGTAGTAAAGATGGACATAAAAACCTAGATTTGCAAAAAATAGTTGTGACTTTAAGATTGAGGAATAAAAGTTTAAGTCAAGTAATATCCGTTGCTAGAGAATGGAACTATATATCACAAAATAGTCTTTCAAATAGTGAGCTAGAATATCAAGTCAAGTATATGTATGAGAAACTTAAAACGGTTAATTTTGGTTGTACTGGTTGTGAGTTTAATAGTGATTGTTGGAATAAAATAGAATCAGATTTTATATATAGTGATGAAGATACTTTGTTCAATATGCCACATAAGCACTCAAAGGATTTGAAATATAAGAATAGGAAAGGGGTTAAAATAATGACTGGTAATCAATTGTTTATCTATAATGTGTTACTTAACAATAAAGATAGAGAATTAAACATAGACGATATAATGGAGCTGATAACCTATAAACGTAAGAAGAAAGTTAAAAACATTGTTATGAGTGAAAAGACATTAAGAGAAACATTAAAAGAACTTCAACATAATGATTATATTACAAAAACAAAAGGTGTTACAAAGCTAGGAATAAAAGATACATACAATGTAAAAGAAGTTAGATGTAATATAGATAAACAATATACTATTAGTTACTTTGTTACCATGGCAGTAATTTGGGGAATAATTTCAACTGAAGAATTAAGATTATATACTCACATGAGATATAAGCAAGATTTATTGGTCAAAGATGATAAAATAAAAGGAAATATATTAAGAATTAATCAAGAGGAATTAGCAAAAGATTTAGGAGTAACACAGCAAAGAATTTCAAATATGATAGAATCTTTATTAGATACTAAAATTTTAGATGTATGGGAAACTAAAATAAATGATAGAGGATTTATGTACTATACATATAGATTAAACAAGTAGATTTTTGATAGGATTAGAATTGATTTTCTAGTCCTATTTTTATGCAAAAAAACTAATTAATAAAAATTTCTTTTGGTAAAATAATTGTACGAGAATTGCAAAAAAAAAATGGCATCAAAGTATTGAAATTAAGCCGTTTTAAAAATTTCTTTTGGTAAAATAATTCTACATATATATGTAGTATATATATATATGTTTTTTAGAGAATGTATAACTAGAATATAGAGCTAGAATATAGAGAATGTATAACTAGAATATAGAGCTAGAATATAGAGAATGTATAACTAGAATATAGAGCTAGAATATAGAGAATGTATAACTAGAATATAGAGCTAGAATATAGAGAATGTATAACTAGAATATAGAGCTAGAATATAGAGAATGTATAACTAGAATATAGAGCTAGAATATAGAGAATGTATAACTAGAATATAGAGCTAGAATCCTAATTAGTAGGTGCTTTTTTAAAACAAGTTAAAAATCAAAAATAGTATTAGTAAGCATTGGAAATGCTAGATTCTAAAATAGAAAAGTAAAAAATTGGTGCACTATCTAAACTTATCTATATCGCTTTTTCCGTCGTTTGGTTCTCTAGTTACGATACAGGGGATATGCTTATATTGAGTTATAGTACTAATCAGTGCTTAATATAGTTAATAAAATTATAGTTACCATAGTTTAGTAACTATGATGTATGTTAGTTAGAAACTTGCATTTCGGCCGGCCAGTGGGCAAGTTGAAAAATTCACAAAAATGTGGTATAATATCTTTGTTCATTAGAGCGATAAACTTGAATTTGAGAGGGAACTTAGATGGTATTTGAAAAAATTGATAAAAATAGTTGGAACAGAAAAGAGTATTTTGACCACTACTTTGCAAGTGTACCTTGTACCTACAGCATGACCGTTAAAGTGGATATCACACAAATAAAGGAAAAGGGAATGAAACTATATCCTGCAATGCTTTATTATATTGCAATGATTGTAAACCGCCATTCAGAGTTTAGGACGGCAATCAATCAAGATGGTGAATTGGGGATATATGATGAGATGATACCAAGCTATACAATATTTCACAATGATACTGAAACATTTTCCAGCCTTTGGACTGAGTGTAAGTCTGACTTTAAATCATTTTTAGCAGATTATGAAAGTGATACGCAACGGTATGGAAACAATCATAGAATGGAAGGAAAGCCAAATGCTCCGGAAAACATTTTTAATGTATCTATGATACCGTGGTCAACCTTCGATGGCTTTAATCTGAATTTGCAGAAAGGATATGATTATTTGATTCCTATTTTTACTATGGGGAAATATTATAAAGAAGATAACAAAATTATACTTCCTTTGGCAATTCAAGTTCATCACGCAGTATGTGACGGATTTCACATTTGCCGTTTTGTAAACGAATTGCAGGAATTGATAAATAGTTAACTAAAAGGAGGTCTACTAATGGAACAATACAAACAAGAATTTATAGAATTTATGGTGGAGAGTAATGTACTTACCTTTGGGGATTTCATAACTAAAAGCGGCAGAAGAACACCATTTTTTATAAATACAGGTAACTACAAGACAGGTAATCAATTAAATAAGTTGGCTAAGTTTTATGCTAAAGCAATATATGATAATTTTGGAGATGATTTTGATATTTTATTTGGGCCTGCATATAAAGGAATACCTTTAAGTGTTTCAGTAGCTATGGCACTTGATAATATTTATGGAATTAATGCAGCTTATTGTTCAAATAGAAAAGAAGTTAAAGATCACGGTGATAAGGGAATACTTCTTGGAGCAAAGCTTGAAGAAGGAGACAGAGTTATAATTGTAGAAGATGTCACAACAGCTGGTACATCAGTATACGAAACAATGCCTATACTTAAATCACAGGCTGAGGTTGATGTAAAGGGAATCATAATATCAGTGGATAGAATGGAAAGAGGTAAGGGAGATAAGAGTGCCTTAACTGAACTTAAAGAAAAGTTTGGATTTAAAACATGTTCTATTGTTACTATGGAAGAGGTAGTAGAATATTTGTATAAGAAAAATATCAATGGCAAAGTAATCATAGATGATAAAATGAAAGATAGAATTAATGAGTACTATAAAGAGTATGGAGTAAAATAGGTTAACTTCAGGTTTGTCTGTAACTAAAAACAAGTATTTAAGCAAAAACATCGTAGAAATACGGTGTTTTTTGTTACCCTAAGTTTAAACTCCTTTTTGATAATCTCATGACCAAAATCCCTTAACGTGAGTTTTCGTTCCACTGAGCGTCAGACCCCGTAGAAAAGATCAAAGGATCTTCTTGAGATCCTTTTTTTCTGCGCGTAATCTGCTGCTTGCAAACAAAAAAACCACCGCTACCAGCGGTGGTTTGTTTGCCGGATCAAGAGCTACCAACTCTTTTTCCGAAGGTAACTGGCTTCAGCAGAGCGCAGATACCAAATACTGTTCTTCTAGTGTAGCCGTAGTTAGGCCACCACTTCAAGAACTCTGTAGCACCGCCTACATACCTCGCTCTGCTAATCCTGTTACCAGTGGCTGCTGCCAGTGGCGATAAGTCGTGTCTTACCGGGTTGGACTCAAGACGATAGTTACCGGATAAGGCGCAGCGGTCGGGCTGAACGGGGGGTTCGTGCACACAGCCCAGCTTGGAGCGAACGACCTACACCGAACTGAGATACCTACAGCGTGAGCTATGAGAAAGCGCCACGCTTCCCGAAGGGAGAAAGGCGGACAGGTATCCGGTAAGCGGCAGGGTCGGAACAGGAGAGCGCACGAGGGAGCTTCCAGGGGGAAACGCCTGGTATCTTTATAGTCCTGTCGGGTTTCGCCACCTCTGACTTGAGCGTCGATTTTTGTGATGCTCGTCAGGGGGGCGGAGCCTATGGAAAAACGCCAGCAACGCGGCCTTTTTACGGTTCCTGGCCTTTTGCTGGCCTTTTGCTCACATGTTCTTTCCTGCGTTATCCCCTGATTCTGTGGATAACCGTATTACCGCCTTTGAGTGAGCTGATACCGCTCGCCGCAGCCGAACGACCGAGCGCAGCGAGTCAGTGAGCGAGGAAGCGGAAGAGCGCCCAATACGCAGGGCCCCCTGCTTCGGGGTCATTATAGCGATTTTTTCGGTATATCCATCCTTTTTCGCACGATATACAGGATTTTGCCAAAGGGTTCGTGTAGACTTTCCTTGGTGTATCCAACGGCGTCAGCCGGGCAGGATAGGTGAAGTAGGCCCACCCGCGAGCGGGTGTTCCTTCTTCACTGTCCCTTATTCGCACCTGGCGGTGCTCAACGGGAATCCTGCTCTGCGAGGCTGGCCGGCTACCGCCGGCGTAACAGATGAGGGCAAGCGGATGGCTGATGAAACCAAGCCAACCAGGAAGGGCAGCCCACCTATCAAGGTGTACTGCCTTCCAGACGAACGAAGAGCGATTGAGGAAAAGGCGGCGGCGGCCGGCATGAGCCTGTCGGCCTACCTGCTGGCCGTCGGCCAGGGCTACAAAATCACGGGCGTCGTGGACTATGAGCACGTCCGCGAGCTGGCCCGCATCAATGGCGACCTGGGCCGCCTGGGCGGCCTGCTGAAACTCTGGCTCACCGACGACCCGCGCACGGCGCGGTTCGGTGATGCCACGATCCTCGCCCTGCTGGCGAAGATCGAAGAGAAGCAGGACGAGCTTGGCAAGGTCATGATGGGCGTGGTCCGCCCGAGGGCAGAGCCATGACTTTTTTAGCCGCTAAAACGGCCGGGGGGTGCGCGTGATTGCCAAGCACGTCCCCATGCGCTCCATCAAGAAGAGCGACTTCGCGGAGCTGGTGAAGTACATCACCGACGAGCAAGGCAAGACCGATCGGGCCCCCTGCA

**Supplementary nucleotide sequence 2 (plasmid pMTL-AMH102)**

**pyrE* left homology arm is shown in blue

**pyrE* right homology arm is shown in purple

*catP selectable marker is shown in Green

CCTGCAGGATAAAAAAATTGTAGATAAATTTTATAAAATAGTTTTATCTACAATTTTTTTATCAGGAAACAGCTATGACCGCGGAAGGGCATTTTCTACTTTCTTCAGGAAAACACAGTGATAGGTATTGTCAGTGTGCAAAACTTTTACAGTATCCTGACAGGGCAAAAGATGTAATAGCAGTTATTGCAGACAAATTAGAGAATGTTGACTATGATAAAATAGTTGGACCTGCAATGGGGGGGATATTAGTTTCCTATGAACTTGCAAGGCAAACGGGCAAACCAGGAATATTTGCTGAAAGGCAAAATGGAAATATGACTATAAGAAGGGGATTTGAAATAAAAGAAGGAGAAAAAATTATAATTTCTGAAGATGTGGTAACTACAGGAAAATCATCTGTAGAGGTTGCTAAGGTAATTCAGGAATTAGGTGGAGAGGTTGTAGGCATATGTTGCATAGTAGACAGAAGAGCAGAAGGTGTCAAAATAGAATATCCAATTTATAGTGCAGTAAAACTTAATATAAACACTTATGATAAGGAAAATTGTCCTATGTGTAAGCAAGGACAAGAATATGTAAAGCCTGGAAGTAGAGTATTCAAATAAGTCGAAAAAATCAATGCACGATGCAGAATTGACAATTAAATTATTAGATTTTAGATTTTTCATAACGCTCACGGAGAAAAATCCACAGAAATTGTGAATTGATTACTTAAAAATAAGCCTATTTCTATAGCACATTAACTGTGTTTTTAGGAATAGGCTTATTTAAGTAAATAGCTTAAATAAGCCTTTCACAATTTTATGGAGTTTTACAAAAAATTATTACTATTTATAAGATTTTTGAGGTATAATAAGTAATGAATTCCTACAATATTATTTATTATATACATGAAAAATTAATAGTGATTTTTTATTGTGTATAATTTTATATAATGGATAATCCTTTGGGAATATTAATCAGGGAATCACCACTTTCTATAATGGGTGATTTAACTTCCCATAGTTATTAATACTAAGTATAGGAGGAGTTTTTTAGTGGGTAAACCAAGTATTTTTAGCAAAGAATATGAAAAGAAAATGCAGCGGCATAAGAGAAATAAAGTTATACTTATTGTAGTATGTTTGATGGCAGCAGTGTTAGTTGTAGTTTACATAAGGGGAGCATTTAGAGATGTAGTAAAGGAAACAAGTAAAGTAAAGAATAATATTGTTTCTGAAAACAAACAGACAAAAAATAATACGGATAGTCAAAAATCATCTCAACCAAATACTCAAACTAAGTCTAAGGATGCAGCAAGTCAGAATTCATATAAAATAAAATTGAGTAGTGGAAAAGATGTAAGTTTAATATATCAAGGTAAGGGTAATGATAAAGTATTTAAGGCAGATAGCAGCGGTACATATGATGTAAGCCCATCAGGTAAGAATGCACTTTTGTTTGATGATAAGTCTCAGAGTATATTGTTAGTGGATGCAAGTGGAAATAAGCAGGATATAACAAATCCACAGTATGTATCTACTACAGGAACTGTAATAGCAAAAGATTCTCAATTAACTAATAATCCAGGATATGTATGGTGTTCTTCACCTAAATTTATAGATGACAATAATATTGCATATGTAAGTCAACTCCCATGGATTGGAAAAACTACAAAATACGTGTGGATAGAAAGCTTGCAGAATAAAAATCATGTTATGGTTCAGAATATAGAAGGTGAAGATATAAAGTTTCAGGGAATAAATGAAAAGGGACTTACGGCCATTGTAGATGGTAAAACTGTTTATTTAACGGCATCAGGTTCCGTTACCCAGTAGGCGCGCCCGCCCTTAAGTCTAAAAATTAGGGGAGATGTAAGGATTTGGGAAAAATAGAAGATGTTATAATCATAAATATGGTATTCGTAGGCTTAAAGTCAAAAAGGAGGTGAAATATAAATAGATTTTTAGCTAAATTAAGTAAGAAATAGGAGGAGATTTATTGAACAAAAAATTAGAAAAACCATTTGTATATAAGAGAGAGTACGATTTGACTGGATATGATGTTGAAATTTTACAAAAATATGAGTTAGAACAAGCAATATATGTTTATGTTGGGAGTAGTTGTGCATATAACATGAGAGCTAGAAGTAGTAAATGGAGATACCATATAAGAACAAATAATAAGTCTATATGTTGTAACATTAAAAATTTTATACATAACTTGGAATTGTTTTATAAAATGGAATTAAAGTTGTCAGATAATATTATTAATGATAAGCTATACTATAGCAATATAGCAGAGTTTGAAGAATTTGAAACACTAGAAAAAGCTAGAGAGGTAGAAAGTACTATTATAAGTCAATATCAATTTTTAGATTCTATAAATCACATGTTAAAACAAAAAATAATTTTATTGAGTAATAAGGATAGTGTGTTAAACATAACTAAAAATGGAAATACAAATTATTTGAAAGTAAAAAATAAATACATAGAAAAACATAAGAACAAGCCAATAATGAGATACCATATCAACTGTCAATTCAATACAGATGGAAGTGTCAAAAGTATTACACAGGAGTTTGAACCAATATTGGAATTAAACAAAAAAAATACCCTAAGCCGACCAAGCAGAGTATTTTTAAAATAATATTTTAAGATAACAACAAAATGAGATAATACTACTAGACAATGACAACTCAACTACCAATTGAGTTTATGGAGCTACCAACTCCAATATCGGTCTAACTGATTAAGTATCTGTAGTTATATAATAATATTGCTATCAATTTTAGCATCTTAACAATATTATTATACATACTAAGCTAAAATTATTCAATAGTTGTAAAAGTTGATTAGTCAATAAGTATATATTTAATGTAGTGTTATCTCTTAAAAAAACTAGATAAGGAGATAATAAATATATGGAACAATTAGATTCAAAATATAAGTTGAAAAAATTTCTAATGGCAGTATTTAGAGATGGTATAGGACAAGGAAATAATCTTATTGATAATGAATATGTTAGAGTATTTCAAAATAATAAAAGTAATAGTAAACAATTAGAACTCGGAGAAGAATTTAAAGAATATAGTAAAACAACTTTTTTTAAAAATATAGATGATATAGTAGAATTTACCTTCGCAAAAAATATTTATTATGAAAATACATTTTTTAACCTATGTACTACTGATGGAAAAGCAGGAACCAATGAAAACTTAATAAATAGATATGCATTAGGATTTGATTTTGACAAAAAAGAATTAGGACAAGGTTTTAATTATAAAGATATAATTAATTTATTTACTAAGATAGGATTACATTATCATATCCTAGTTGATAGTGGAAATGGATTCCATGTTTATGTGCTAATTAATAAAACTAATAACATTAAGTTAGTATCAGAAGTTACAAATACATTAATAAATAAATTGGGTGCAGATAAACAAGCAAATTTATCTACTCAAGTATTAAGAGTACCTTATACATATAATATTAAAAATACTACTAAACAAGTAAAAATAATACACCAAGACAAAAATATATATAGATATGACATAGAAAAGTTAGCTAAAAAATATTGCAAAGATGTAAAAACAGTAGGTAATACTAATACAAAATATATATTAGATAGTAAGCTACCAAATTGTATAGTAGATATTTTAAAAAATGGTAGTAAAGATGGACATAAAAACCTAGATTTGCAAAAAATAGTTGTGACTTTAAGATTGAGGAATAAAAGTTTAAGTCAAGTAATATCCGTTGCTAGAGAATGGAACTATATATCACAAAATAGTCTTTCAAATAGTGAGCTAGAATATCAAGTCAAGTATATGTATGAGAAACTTAAAACGGTTAATTTTGGTTGTACTGGTTGTGAGTTTAATAGTGATTGTTGGAATAAAATAGAATCAGATTTTATATATAGTGATGAAGATACTTTGTTCAATATGCCACATAAGCACTCAAAGGATTTGAAATATAAGAATAGGAAAGGGGTTAAAATAATGACTGGTAATCAATTGTTTATCTATAATGTGTTACTTAACAATAAAGATAGAGAATTAAACATAGACGATATAATGGAGCTGATAACCTATAAACGTAAGAAGAAAGTTAAAAACATTGTTATGAGTGAAAAGACATTAAGAGAAACATTAAAAGAACTTCAACATAATGATTATATTACAAAAACAAAAGGTGTTACAAAGCTAGGAATAAAAGATACATACAATGTAAAAGAAGTTAGATGTAATATAGATAAACAATATACTATTAGTTACTTTGTTACCATGGCAGTAATTTGGGGAATAATTTCAACTGAAGAATTAAGATTATATACTCACATGAGATATAAGCAAGATTTATTGGTCAAAGATGATAAAATAAAAGGAAATATATTAAGAATTAATCAAGAGGAATTAGCAAAAGATTTAGGAGTAACACAGCAAAGAATTTCAAATATGATAGAATCTTTATTAGATACTAAAATTTTAGATGTATGGGAAACTAAAATAAATGATAGAGGATTTATGTACTATACATATAGATTAAACAAGTAGATTTTTGATAGGATTAGAATTGATTTTCTAGTCCTATTTTTATGCAAAAAAACTAATTAATAAAAATTTCTTTTGGTAAAATAATTGTACGAGAATTGCAAAAAAAAAATGGCATCAAAGTATTGAAATTAAGCCGTTTTAAAAATTTCTTTTGGTAAAATAATTCTACATATATATGTAGTATATATATATATGTTTTTTAGAGAATGTATAACTAGAATATAGAGCTAGAATATAGAGAATGTATAACTAGAATATAGAGCTAGAATATAGAGAATGTATAACTAGAATATAGAGCTAGAATATAGAGAATGTATAACTAGAATATAGAGCTAGAATATAGAGAATGTATAACTAGAATATAGAGCTAGAATATAGAGAATGTATAACTAGAATATAGAGCTAGAATATAGAGAATGTATAACTAGAATATAGAGCTAGAATCCTAATTAGTAGGTGCTTTTTTAAAACAAGTTAAAAATCAAAAATAGTATTAGTAAGCATTGGAAATGCTAGATTCTAAAATAGAAAAGTAAAAAATTGGTGCACTATCTAAACTTATCTATATCGCTTTTTCCGTCGTTTGGTTCTCTAGTTACGATACAGGGGATATGCTTATATTGAGTTATAGTACTAATCAGTGCTTAATATAGTTAATAAAATTATAGTTACCATAGTTTAGTAACTATGATGTATGTTAGTTAGAAACTTGCATTTCGGCCGGCCAGTGGGCAAGTTGAAAAATTCACAAAAATGTGGTATAATATCTTTGTTCATTAGAGCGATAAACTTGAATTTGAGAGGGAACTTAGATGGTATTTGAAAAAATTGATAAAAATAGTTGGAACAGAAAAGAGTATTTTGACCACTACTTTGCAAGTGTACCTTGTACCTACAGCATGACCGTTAAAGTGGATATCACACAAATAAAGGAAAAGGGAATGAAACTATATCCTGCAATGCTTTATTATATTGCAATGATTGTAAACCGCCATTCAGAGTTTAGGACGGCAATCAATCAAGATGGTGAATTGGGGATATATGATGAGATGATACCAAGCTATACAATATTTCACAATGATACTGAAACATTTTCCAGCCTTTGGACTGAGTGTAAGTCTGACTTTAAATCATTTTTAGCAGATTATGAAAGTGATACGCAACGGTATGGAAACAATCATAGAATGGAAGGAAAGCCAAATGCTCCGGAAAACATTTTTAATGTATCTATGATACCGTGGTCAACCTTCGATGGCTTTAATCTGAATTTGCAGAAAGGATATGATTATTTGATTCCTATTTTTACTATGGGGAAATATTATAAAGAAGATAACAAAATTATACTTCCTTTGGCAATTCAAGTTCATCACGCAGTATGTGACGGATTTCACATTTGCCGTTTTGTAAACGAATTGCAGGAATTGATAAATAGTTAACTTCAGGTTTGTCTGTAACTAAAAACAAGTATTTAAGCAAAAACATCGTAGAAATACGGTGTTTTTTGTTACCCTAAGTTTAAACTCCTTTTTGATAATCTCATGACCAAAATCCCTTAACGTGAGTTTTCGTTCCACTGAGCGTCAGACCCCGTAGAAAAGATCAAAGGATCTTCTTGAGATCCTTTTTTTCTGCGCGTAATCTGCTGCTTGCAAACAAAAAAACCACCGCTACCAGCGGTGGTTTGTTTGCCGGATCAAGAGCTACCAACTCTTTTTCCGAAGGTAACTGGCTTCAGCAGAGCGCAGATACCAAATACTGTTCTTCTAGTGTAGCCGTAGTTAGGCCACCACTTCAAGAACTCTGTAGCACCGCCTACATACCTCGCTCTGCTAATCCTGTTACCAGTGGCTGCTGCCAGTGGCGATAAGTCGTGTCTTACCGGGTTGGACTCAAGACGATAGTTACCGGATAAGGCGCAGCGGTCGGGCTGAACGGGGGGTTCGTGCACACAGCCCAGCTTGGAGCGAACGACCTACACCGAACTGAGATACCTACAGCGTGAGCTATGAGAAAGCGCCACGCTTCCCGAAGGGAGAAAGGCGGACAGGTATCCGGTAAGCGGCAGGGTCGGAACAGGAGAGCGCACGAGGGAGCTTCCAGGGGGAAACGCCTGGTATCTTTATAGTCCTGTCGGGTTTCGCCACCTCTGACTTGAGCGTCGATTTTTGTGATGCTCGTCAGGGGGGCGGAGCCTATGGAAAAACGCCAGCAACGCGGCCTTTTTACGGTTCCTGGCCTTTTGCTGGCCTTTTGCTCACATGTTCTTTCCTGCGTTATCCCCTGATTCTGTGGATAACCGTATTACCGCCTTTGAGTGAGCTGATACCGCTCGCCGCAGCCGAACGACCGAGCGCAGCGAGTCAGTGAGCGAGGAAGCGGAAGAGCGCCCAATACGCAGGGCCCCCTGCTTCGGGGTCATTATAGCGATTTTTTCGGTATATCCATCCTTTTTCGCACGATATACAGGATTTTGCCAAAGGGTTCGTGTAGACTTTCCTTGGTGTATCCAACGGCGTCAGCCGGGCAGGATAGGTGAAGTAGGCCCACCCGCGAGCGGGTGTTCCTTCTTCACTGTCCCTTATTCGCACCTGGCGGTGCTCAACGGGAATCCTGCTCTGCGAGGCTGGCCGGCTACCGCCGGCGTAACAGATGAGGGCAAGCGGATGGCTGATGAAACCAAGCCAACCAGGAAGGGCAGCCCACCTATCAAGGTGTACTGCCTTCCAGACGAACGAAGAGCGATTGAGGAAAAGGCGGCGGCGGCCGGCATGAGCCTGTCGGCCTACCTGCTGGCCGTCGGCCAGGGCTACAAAATCACGGGCGTCGTGGACTATGAGCACGTCCGCGAGCTGGCCCGCATCAATGGCGACCTGGGCCGCCTGGGCGGCCTGCTGAAACTCTGGCTCACCGACGACCCGCGCACGGCGCGGTTCGGTGATGCCACGATCCTCGCCCTGCTGGCGAAGATCGAAGAGAAGCAGGACGAGCTTGGCAAGGTCATGATGGGCGTGGTCCGCCCGAGGGCAGAGCCATGACTTTTTTAGCCGCTAAAACGGCCGGGGGGTGCGCGTGATTGCCAAGCACGTCCCCATGCGCTCCATCAAGAAGAGCGACTTCGCGGAGCTGGTGAAGTACATCACCGACGAGCAAGGCAAGACCGATCGGGCCC

**References**

1. **Williams DR, Young DI, Young M.** 1990. Conjugative plasmid transfer from *Escherichia coli* to *Clostridium acetobutylicum*. Journal of General Microbiology **136:**819-826.

2. **Weisburg WG, Barns SM, Pelletier DA, Lane DJ.** 1991. 16S ribosomal DNA amplification for phylogenetic study. Journal of Bacteriology **173:**697-703.

3. **Heap JT, Pennington OJ, Cartman ST, Minton NP.** 2009. A modular system for *Clostridium* shuttle plasmids. Journal of Microbiological Methods **78:**79-85.

4. **Heap JT, Pennington OJ, Cartman ST, Carter GP, Minton NP.** 2007. The ClosTron: A universal gene knock-out system for the genus *Clostridium*. Journal of Microbiological Methods **70:**452-464.
